# Genome-Wide Identification of *bZIP* Family Genes Involved in Drought and Heat Stresses in Strawberry (*Fragaria vesca*)

**DOI:** 10.1155/2017/3981031

**Published:** 2017-04-11

**Authors:** Xiao-Long Wang, Xinlu Chen, Tian-Bao Yang, Qunkang Cheng, Zong-Ming Cheng

**Affiliations:** ^1^College of Horticulture, Nanjing Agricultural University, Nanjing 210095, China; ^2^Department of Plant Sciences, University of Tennessee, Knoxville, TN 37996-4560, USA; ^3^Food Quality Laboratory, Beltsville Agricultural Research Center, Agricultural Research Service, United States Department of Agriculture, Beltsville, MD 20705, USA; ^4^Department of Entomology and Plant Pathology, University of Tennessee, Knoxville, TN 37996-4560, USA

## Abstract

Basic leucine zipper (bZIP) genes are known to play a crucial role in response to various processes in plant as well as abiotic or biotic stress challenges. We have performed an identification and characterization of 50 *bZIP* genes across the woodland strawberry (*Fragaria vesca*) genome, which were divided into 10 clades according to the phylogenetic relationship of the strawberry bZIP proteins with those in *Arabidopsis* and rice. Five categories of intron patterns were observed within basic and hinge regions of the bZIP domains. Some additional conserved motifs have been found with the group specificity. Further, we predicted DNA-binding specificity of the basic and hinge regions as well as dimerization properties of leucine zipper regions, which was consistent with our phylogenetic clade and classified into 20 subfamilies. Across the different developmental stages of 15 organs and two types of fruits, the clade A *bZIP* members showed different tissue-specific expression patterns and the duplicated genes were differentially regulated, indicating a functional diversification coupled with the expansion of this gene family in strawberry. Under normal growth conditions, *mrna11837* and *mrna30280* of clade A showed very weak expression levels in organs and fruits, respectively; but higher expression was observed with different set of genes following drought and heat treatment, which may be caused by the separate response pathway between drought and heat treatments.

## 1. Introduction

The transcription factors containing bZIP domain [1, 2] constitute one of the largest gene families in plants. The common feature, bZIP domain with length ranging from 60 to 80 amino acids (aa), includes two unique structures, a highly conserved DNA-binding basic and hinge region and a relatively diversified leucine zipper region [3]. The basic and hinge region consists of 18 aa residues with an invariant motif (N-X_7_-R/K-X_9_). In contrast, the downstream leucine zipper region is made up of heptad leucine repeats, a repeated pattern of several amino acids, or other hydrophobic amino acids, such as Ile, Phe, and Met [4]. The leucine zipper region is located exactly 9 amino acids downstream from the C-terminal of the basic region. It has been proved that the leucine zipper region is more favorable to constitute an amphipathic-helix with two helical turns within each heptad, because of its special amino acid composition [1, 2].

To date, numerous bZIP members have been investigated and characterized by various eukaryotic genomes. Previous studies have identified 17 *bZIP* genes in *Saccharomyces cerevisiae* [5], 89 in rice [2], 56 in human [6], 131 in soybean [7], 125 in maize [4], 75 in *Arabidopsis* [8], 92 in *Sorghum* [9], 49 in *Ricinus communis* L. [10], and 55 in grapevine [11]. Considerable evidence showed that plant bZIP proteins play crucial roles in various aspects of biological processes, including organ and tissue developments [12, 13], embryogenesis [14], seed maturation [15], cell elongation [16, 17], and nitrogen/carbon and energy metabolism [18–20]. In the recent years, increasing evidence has also indicated that bZIP proteins take part in the regulation of plants' response to abiotic and biotic stresses, including phytohormone abscisic acid (ABA) signaling [21, 22], osmosis [19, 23], drought [24, 25], high salinity [26, 27], cold stresses [7, 28], pathogen defense [29, 30], and light irradiation [31, 32].

Although the bZIP transcription factor family in strawberry (*F. vesca*), apple, and peach has been identified [33], only information on the evolutionary characterization of *bZIP* genes in Rosaceae was provided. Limited evidence is available regarding basic annotation of structural and functional features in the bZIP family among the economically important strawberry. The cultivated strawberry (*F. × ananassa*) is a model plant for nonclimacteric fruit species. *F. × ananassa* derived from 4 diploid ancestors has a relatively complex octaploid genome harboring 56 chromosomes (2n = 8x = 56). In contrast, our experiment system *F. vesca*, the diploid woodland strawberry, has been sequenced, which offers substantial advantages for molecular and physiological studies [34]. In this research, we performed comprehensive studies on expansion pattern, structural annotation, DNA-binding site specificity, dimerization properties, and expression levels in different stages and abiotic treatments to shed light on *bZIP* genes in *F. vesca*.

## 2. Materials and Methods

### 2.1. Identification of *bZIP* Genes in Woodland Strawberry

To generate a comprehensive list of *F. vesca bZIP* genes, *F. vesca v1.1* genome was downloaded from Phytozome (https://phytozome.jgi.doe.gov/pz/portal.html). Firstly, a HMM profile of the bZIP domain (PF00170) was downloaded from Pfam (http://pfam.xfam.org/). Secondly, HMMER analysis was performed to search all *F. vesca* protein sequences and extracted the corresponding coding DNA sequence (CDS) and amino acid sequences. Thirdly, all of the putative bZIP domains were manually verified (*E* value < 1.0) within Pfam and SMART (http://smart.embl-heidelberg.de/) to confirm their presence and completeness of bZIP domain. Molecular weight, instability index, isoelectric points, aliphatic index, and grand average of hydropathicity (GRAVY) of candidate FvbZIP proteins were detected in ExPASy ProtParam server (http://web.expasy.org/protparam/).

### 2.2. Phylogenetic Analysis and Structures of *FvbZIP* Gene

Phylogenetic analysis was used for classifying *FvbZIP* genes into ten clades [33]. The amino acid sequences of bZIP domains located in bZIP proteins from strawberry, *A. thaliana* (AtbZIP) [8], and rice (OsbZIP) [2] were used to generate a phylogenetic tree through ClustalW alignment and the unrooted Neighbor-joining (N-J) method using MEGA 6.0 [35]. N-J analysis with pairwise deletion and the Jones-Taylor-Thornton (JTT) model was performed using the 1000 replicate bootstraps to support for each node in phylogenetic tree. The exon-intron organizations with intron phase in *FvbZIP*s were performed by alignment within CDS sequence and corresponding genomic sequence and presented by GSDS 2.0 (http://gsds.cbi.pku.edu.cn/).

### 2.3. Conserved Motif Identification of FvbZIP Proteins

The motif distribution of *FvbZIP* proteins were extracted by MEME (http://meme.nbcr.net/meme/), and parameters are set as follows: the largest number of discovered and conserved motifs set 12 and motif width was from 30 to 50 aa. The remaining parameter settings were kept default.

### 2.4. Dimerization Properties of FvbZIP Proteins

To describe the foundation of the dimerization stability and speculate the dimerization specificity of 50 FvbZIP members, we named special amino acid residue positions as *g*, *a*, *b*, *c*, *d*, *e*, and *f* to put in order of each heptad on the basis of standard nomenclature for the seven heptad repeats [36] (see Supplementary Figure S4 available online at https://doi.org/10.1155/2017/3981031). The first heptad was manually arranged from four amino acid residues (corresponding amino acid located at *g* position in the first heptad) before appearance of the first leucine in the bZIP domain. Both boundaries of N-terminal and C-terminal within the FvbZIP leucine zipper region have been the same as applied to bZIP proteins in *Arabidopsis* [37].

### 2.5. Plant Materials and Treatments

*F. vesca* (National Clonal Germplasm Repository accession number PI664444) were cultivated in growth chamber with 22°C in a 12/12 h dark/light photoperiod. Strawberry plants were transferred into 42°C chamber for 0, 1, 6, 12, and 24 h posttreatment (hpt) for heat stress treatment. Drought treatment was simulated by depleting water after fully watering plants and sampled at 0 (water content on normalized soil is 83.96%), 48 (79.18%), 96 (63.41%), and 144 (36.78%) hpt. The strawberry plants were rewatered followed 144 h (74.78%) of drought treatment and sampled again 48 h later. All of the experiments were performed three times.

### 2.6. Quantitative RT-PCR Analysis

Transcriptional changes of *FvbZIP* genes responding to drought and heat treatments were determined by quantitative RT-PCR analysis on Stratagene Mx3000P Real-Time PCR system using SYBR® Premix Ex Taq™ (TaKaRa, Japan). The PCR amplification conditions were as follows: 95°C 10 min, 40 cycles of 15 sec at 95°C, and 1 min at 60°C; at the end, the melting curve analysis was executed for verifying the specificity of the primer with the following stage: 95°C for 15 sec, 60°C for 1 min, and 95°C for 15 sec. The relative gene expression was determined using an interspacer 18S-26S strawberry gene as an endogenous control gene [38]. The relative expression levels of the target genes were calculated by the 2^−ΔΔCt^ method [39]. Each specific primer pair was listed in Supplementary Table S1. Three independent biological replications were performed at each treatment point.

RPKM (reads per kilobase of exon per million mapped reads) of *FvbZIP* genes in different tissues and early stage fruit development were directly downloaded from available database (http://bioinformatics.towson.edu/strawberry/) (Table S5), which reflects mRNA levels. The expression levels (RPKM) for *FvbZIP* genes in yellow and red fruits of strawberry were from Tianbao Yang's lab in USDA-ARS. To compare the differential expression of bZIP genes (Table S5), the expression level of each gene in the different stages was read and normalized using multiexperiment viewer (MeV) software [40].

## 3. Results and Discussion

### 3.1. Identification and Characterization of Strawberry bZIP Family

A total of 50 *FvbZIP* genes were identified in strawberry genome in Phytozome. The characteristic parameters of all predicted FvbZIP proteins are listed in Table 1, including chromosome location, protein length, molecular weight, theoretical pI, instability index, aliphatic index, and grand average of hydropathicity (GRAVY). The length of amino acids of *FvbZIPs* is from 114 (mrna26148) to 1106 (mrna03778) aa with an average of 402.54 aa. Compared to those in *Arabidopsis* [8] (harboring 75 members with an average 321 aa in length) and rice [2] (harboring 89 members with an average 311 aa in length), strawberry has 50 bZIP members with longer average length than these two plants. After divergence of monocots and eudicots, lower frequency of bZIP candidates' evolution occurred in eudicots than monocots [9], which may explain the fact that strawberry and *Arabidopsis* have fewer bZIP members than rice. The predicted molecular weights of *FvbZIP* proteins range from 13.51 (mrna26148) to 122.71 (mrna03778) kDa, with pI from 4.73 (mrna29159) to 9.56 (mrna11666) (Table 1).

### 3.2. Expansion Pattern of bZIP Family in Strawberry, *Arabidopsis*, and Rice

To survey the extent of species-specific expansion of the bZIP genes in strawberry, *Arabidopsis*, and rice, we performed a joint phylogenetic analysis of strawberry, *Arabidopsis*, and rice bZIP proteins. As shown in *Arabidopsis* and rice analysis [2, 8], the joint phylogenetic tree of three species grouped all bZIPs in ten distinct clades (A, B, C, D, E, F, G, H, I, and S) (Supplemental Figure S1A). Six bZIP genes in strawberry, *Arabidopsis*, and rice could not be conclusively mapped to any clade and was renamed U. We investigated some specific nodes which led to strawberry-, *Arabidopsis*-, and rice-specific classes within clades (red circle on node in Supplemental Figure S1A). As these nodes located in specific classes among strawberry, *Arabidopsis*, and rice represent the divergence point in the evolution and indicate the most recent common ancestors (MRCAs) prior to division, we found that some bZIP genes possibly have been present in the MRCAs of strawberry, *Arabidopsis*, and rice, but one or two species-specific *bZIP*s were lost in strawberry, *Arabidopsis*, or rice. Six classes contained only strawberry *bZIP* genes (Supplemental Figure S1A, red arrows), fifteen classes contained only *Arabidopsis bZIP* genes (Supplemental Figure S1A, green arrows), and thirty classes only contained rice *bZIP* genes (Supplemental Figure S1A, yellow arrows), suggesting that gene loss event has occurred in those classes in other species. The interspecies classes revealed that a parallel evolution of *bZIP* genes happened in three plant species and the orthologous bZIP proteins were observed to play a similar role [41]. The number of classes illustrated a fact that at least 85 ancestral *bZIP* genes existed before the strawberry, *Arabidopsis*, and rice split. After splitting from the MRCA, strawberry has lost the largest number of *bZIP* genes, followed by *Arabidopsis* and rice (Supplemental Figure S1B). A wide range interspecies comparison of strawberry, *Arabidopsis*, and rice revealed the species specific and nonspecific proteins.

### 3.3. Gene Structure of FvbZIP Genes

**Figure 1:**
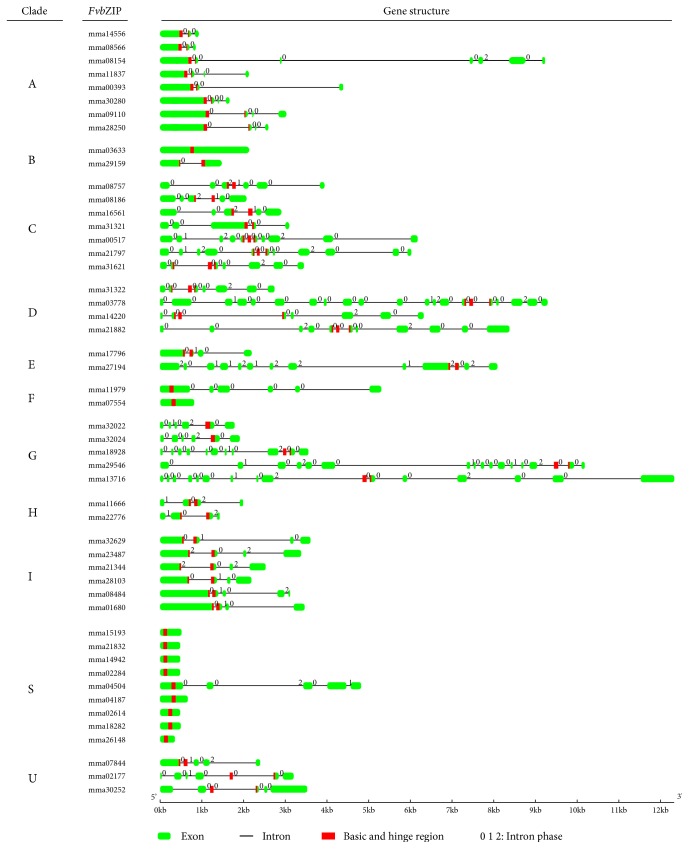
Exon/intron organization of *FvbZIP* genes was depicted for each group. The exons and introns are represented by green boxes and black lines, respectively. The red boxes denote the basic and hinge region of bZIP domain. The numbers above line denote the splicing phase. The numbers “0” and “2” denote different splicing phases, “0” means splicing occurred after the third nucleotide of the codon, and “2” means splicing occurred after the second nucleotide.

**Figure 2:**
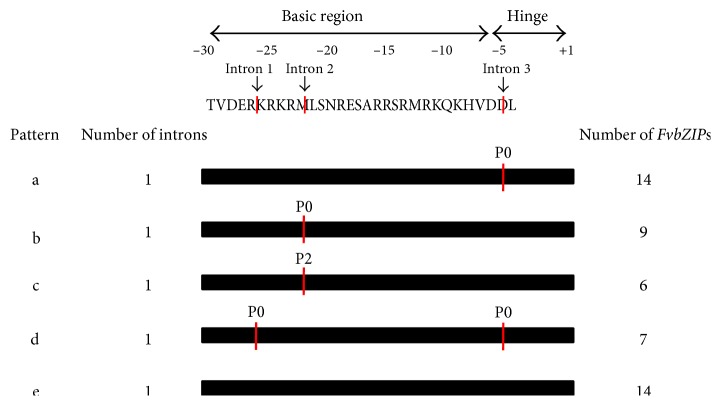
Intron patterns (a–e) within the basic and hinge regions of bZIP proteins. P0 and P2 stand for the intron splicing phase. P0 means splicing occurred after the third nucleotide of the codon, and P2 means splicing occurred after the second nucleotide. The black bars represent the sequence of the basic and hinge regions. The vertical lines denote the positions of intron splicing phases.

In order to observe the structure of the *FvbZIP* genes, we performed an analysis of the number and distribution of exon-intron, which are recorded to play key roles in gene family evolution and further promote to comprehend the emergence and evolution of a given gene [42, 43]. As shown in Figure 1, 10 (20%) of total *FvbZIP* genes exhibit intronless, which occurs exclusively in group S, accounting for 88.9%. About 20% of peach, 19.1% of rice, 15.3% of maize, and 20.4% of castor bean bZIP genes were predicted to be intronless [2, 4, 10, 44]. Among *FvbZIP* genes containing introns, the number of introns in the open reading frame (ORF) varied from one (*mrna29159*) to 20 (*mrna03778*), indicating a great diversity in the *FvbZIP* members (Figure 1). According to number, position, and splicing phase of the introns located in *FvbZIP* genes, the considerably conserved basic and hinge regions within the bZIP domain were divided into five patterns (a, b, c, d, and e) (Figure 2; Supplemental Figure S2). A previous study has showed that patterns of intron positions and splicing phases located at the bZIP domain regions were widely regarded as a promoter to understand homology and evolution of *bZIP* genes [2]. Based on intron location relative to the codon, introns can be classified into three patterns. Introns that do not interrupt the codons, between the first and second bases of codon and between the second and third bases of the codon, are termed phase 0 (P0), phase 1 (P1), and phase 2 (P2), respectively [45].

Patterns a (including 14 *FvbZIP*s) and e (including 14 *FvbZIP*s) were the most widespread (Figure 2). Pattern a had an intron in P0 within the hinge region behind the position −6 (Q). Both patterns b and c had one intron each followed the position −22 (R) located at the basic region, but the phase was different. Pattern b had an intron in P0, whereas pattern c had an intron in P2. Pattern d with two introns (both located at P0), one followed position −26 (K) of the basic region and the other one located at the hinge region at position −5, was interrupted by Lys and Ala. Pattern e had no intron distribution neither in the basic nor hinge region. Ten (71.2%) *FvbZIP*s within pattern g were intronless, while the other 4 had introns located at the outside of their basic and hinge regions (Figures 1 and 2). In particular, the position and phase were observed to be highly conserved when there was an intron interrupting in the hinge region. However, positions of introns located in the basic region were variably distributed in P0 or P2 (Figure 2; Supplemental Figure S2). The similar intron distribution and splicing patterns were observed in the bZIP domain regions of rice, castor bean, and maize, which declares that the intron pattern within the basic and hinge regions of *bZIP* genes were highly conserved in plants [2, 4, 10].

Though the lengths of intron were variable (Figure 1), most members clustered in the same clade classified above (Supplemental Figure S1) exhibited the same or similar intron number and splicing phase. For instance, same splicing phases and gene structures were observed in four *FvbZIP* members within clade A and eight members within clade S; five members in clade I also showed a similar splicing phase and gene structure. The intron pattern a was shared in the whole members in clade A, clade S consisted of the most intronless members showed the intron pattern e, members in clade C showed intron pattern c, and clade D (except for mrna31321) shared pattern d. In conclusion, the conserved splicing phases and gene structures in each clade were in agreement with the classification of *FvbZIP*s in phylogenetic analysis.

### 3.4. Conserved Motifs of FvbZIP Proteins

**Figure 3:**
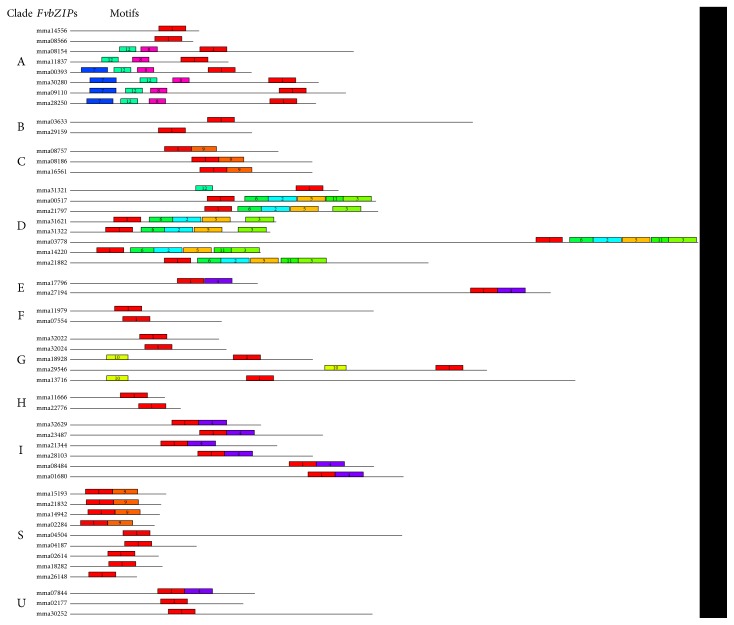
Summery for the distribution of conserved motifs identified from FvbZIP proteins by each clade given separately. Each motif is represented by a number in colored box. See Supplementary Table S1 for detailed motif information.

Besides the bZIP domain, some of the other multiple conserved motifs had been detected in *Arabidopsis*, rice, maize, caster bean, and peach [2, 4, 8, 10, 44]. As shown in Figure 3, a total of 12 conserved motifs were identified in *FvbZIP* genes. The consensus sequence and the amino acid width of those conserved motifs are given in Supplemental Table S2. It can be observed that some motifs are shared by several clades, such as motif 9 which was located in clades C and S, motif 4 shared by clades E and I, and motif 12 which was present in clades A and D. Furthermore, majority of conserved motifs appear in a specific clade, for instance, motifs 2, 3, 5, 6, and 11 in clade D; motifs 7 and 8 in clade A; and motif 10 in clade G (Figure 3). It is a proved hypothesis that the group-specific conserved motifs promote to decide specific functions of *FvbZIP* genes in each clade and foundation for the functional divergences between members from different clades [46–48]. The conserved motif analysis was further proved to be consistent with phylogenetic relationship and classification of *FvbZIP* genes.

Notably, motifs 2, 9, and 10 were identified as DOG1 (delay of germination), bZIP C, and MFMR (multifunctional mosaic region) according to Pfam databases, respectively. The previous study has found DOG1-corresponding gene to be related to seed dormancy in a form of quantitative trait locus [49]. It has been suggested that N-terminal half of MFMR motif is rather rich in proline residues and has been termed the PRD [50], and some of these motifs may play a key role in regulating protein-protein interactions [51]. Motif 10 with a Pro-rich domain was supposed to be involved in mediating protein-protein interactions [51]. Motifs 7, 8, and 12 represented potential casein kinase II (CKII) phosphorylation sites (S/TxxD/E) [52], presented as T[FL]DE, [TQS][LM][GC][GDE], and [ST]AE[EA]; TLGE and TLE[DE]; and TVDE, respectively. These CKII phosphorylation sites were considered to be involved in ABA-mediate responses [26, 53]. A part of these three motifs also contains another phosphorylation site (R/KxxS/T) associated with Ca^2+^-dependent protein kinase, presented as R[EQ][ANST]S and GK[DNP][FL][GS], RQ[PQ]T, and RQG[SG] and [AR][TAP]LS in motifs 7, 8, and 12, respectively. Motif 4 with a glutamine-rich domain has been proved to play an important role in transcriptional activation [54]. We can speculate a similar function of bZIP proteins due to conserved motifs shared by *Arabidopsis*, rice, castor bean, and maize, while the roles of other conserved motifs found in FvbZIP proteins are not yet clear.

### 3.5. DNA-Binding Site Specificity of FvbZIP Proteins

DNA-binding specificity owed by bZIP proteins is determined by certain key amino acids located at the most conserved basic and hinge region of the bZIP domain, and these two regions have a direct interaction with DNA *cis*-elements [55, 56]. The annotation and classification of amino acids located at the basic and hinge region were characterized by diagnostic sequence structure in *Arabidopsis* and rice [2, 8], which indicates that some highly conserved amino acids are present in each clade (Supplementary Figure S3). The first leucine of the leucine zipper region was designed as +1, and C-terminal amino acid of the hinge region was numbered −1 [55]. It is proved that some functional replacements on invariant sites (−18 and −10) by other amino acids will lead to new DNA-binding specificities [57]. In the strawberry bZIP family, these replacements infrequently happened and just occurred in one group. *mrna30252* in clade U with a hydrophobic Ile (Isoleucine) residue at position −10 replaced arginine/lysine, indicating that perhaps they are not able to bind DNA or might hold a unique DNA-binding specificity. Furthermore, we can speculate the structural characteristics and DNA-binding specificity of FvbZIP proteins in a clade model, as listed in Supplementary Table S3. Expression level of a particular gene is sometimes determined by binding affinity to specific sites of promoters of genes, which is resulted of variations in their target sites in promoters of different genes [2]. Computational discovery and further experimental verification of DNA binding ability would promote to identify which genes are selectively activated by various *FvbZIP* genes.

### 3.6. Dimerization Properties of FvbZIP Proteins

**Figure 4:**
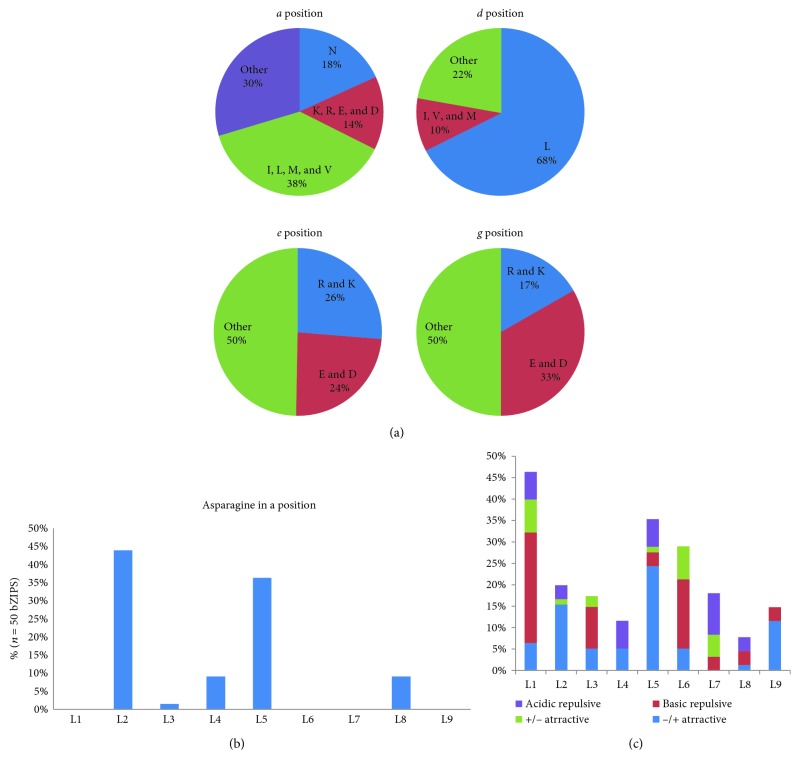
Prediction of the dimerization properties of FvbZIP proteins. (a) Pie charts depicting the frequency of different amino acids at the *a*, *d*, *e*, and *g* positions of the leucine zippers of all FvbZIP proteins (see Supplementary Figure S3 for the amino acid positions within the leucine zipper regions). (b) Histogram of the frequency of the Asn residues present at the *a* position of each heptad within leucine zipper for all bZIP proteins. (c) Histogram of the frequency of attractive or repulsive *g* ↔ *e*′ pairs per heptad within leucine zipper for all FvbZIP proteins.

The function of bZIP proteins requires dimerization between parallel coiled-coil constructions [58]. Amino acids present at positions *a*, *d*, *e*, and *g* near the leucine zipper interface play an important role in regulating oligomerization of leucine zipper domain as well as specificity and stability of dimerization [4]. Thus, we performed detailed characterization of the amino acids located at positions *a*, *d*, *e*, and *g* to predict their functions. The length of the FvbZIP leucine zipper is variable to range from two to nine heptads. About 18% of amino acids located at position *a* were Asns (asparagine) in 41 FvbZIP proteins (Figure 4(a)), which is similar to both 16% in humans and *H. sapiens*, but lesser than observed in *Arabidopsis* (22%) [37], rice (23%) [2], maize (22%) [4], and castor bean (26%) [10]. The highest frequency of Asn at position *a* was displayed in the second heptad (accounting for 44%) followed by the fifth heptad (accounting for 36%) (Figure 4(b)), which is also observed earlier for rice and *Arabidopsis* [2, 37]. Since Asns can form a more stable N-N interaction at the *a* ↔ *a*′ position than the other *a* position amino acids, high frequency of Asn at the *a* position promote to create homodimerizing Leu zippers among the bZIP family [59]. This result raises the possibility that a majority of FvbZIPs prefer to homodimerization. It is observed that minority of charged amino acids (R, K, E, and D) displayed in position *a*, which may contribute to form hetero-dimerization.

Amino acids located at positions *a* and *d* are generally hydrophobic residues and pack on the surface of “knobs and holes” pattern to establish a hydrophobic core area that is important to homo-/hetero-dimerization [60]. The frequency of the conserved Leu responsible for dimer stability at *d* position, was found in 68% of 50 FvbZIP proteins. The abundance of other hydrophobic amino acid (including I, V, and M) at *d* position was comparable in FvbZIPs (10%, Figure 4(a)), *Arabidopsis* (19%), and rice (17%). The Leu at position *d* is a type of highly conserved and stable aliphatic amino acid residue [60], which is critical for keeping the dimerization stability. Compared with the AtbZIP proteins (the proportion of Leu at *d* position accounts for 56%) [37] and RcbZIP proteins (56%) [10], FvbZIPs showed more abundant Leu located at *d* position, which suggests that strawberry genes probably hold a stronger stability dimerization on Leu zipper than *Arabidopsis*.

It is observed that the abundance of charged amino acids displayed at *e* and *g* positions, such as acidic amino acids (including E and D) and basic amino acids (including R and K), accounts for 50% and 50%, respectively (Figure 4(a)). Compared to *Arabidopsis* (charged amino acids at *e* and *g* positions were, respectively, accounting for 41% and 53%), the frequencies of charged amino acids located at *e* position was higher but was lower in strawberry at *g* position. The charged amino acids located at positions *g* and *e* were replaced by alanine can result in creating tetramers instead of dimmers [61]. A greater number of charged amino acids (containing E, D, R, and K) located at *e* and *g* positions were found to flank the dimerization interface, which are thought to interact electrostatically to form salt bridges mediating the dimerization and determining the dimerization specificity as well as stability [62].

As dimerization properties of FvbZIP proteins were governed by different contributions of charged amino acids located at *e* and *g* positions, we calculated the presence of attractive and repulsive *g* ↔ *e*′ pairs in each heptad of strawberry Leu zippers (Supplementary Figure S4) and displayed corresponding frequency histogram in Figure 4(c). Four groups (basic repulsive, acidic repulsive, +/− attractive, and −/+ attractive) were found in complete *g* ↔ *e*′ pairs according to both the *g* and following *e* position amino acids are charged, while incomplete *g* ↔ *e*′ pairs were defined as only one charged position in *g* or *e* [10]. The highest frequency of the complete *g* ↔ *e*′ pairs was observed to locate at the first heptad for 46%, majority of which was found to be the basic repulsive *g* ↔ *e*′ pair. In following three heptads, the frequencies of the complete *g* ↔ *e*′ pairs showed a sharp decline. The frequency of complete *g* ↔ *e*′ pairs appeared to be increased in the fifth heptad, especially on the −/+ attractive gene pairs. Moreover, attractive *g* ↔ *e*′ (including +/− attractive and −/+ attractive) pairs were found most frequently within the second, fifth, and ninth heptad. It is observed that the attractive *g* ↔ *e*′ pairs had no tend to form homo- or hetero-dimerization, while the repulsive *g* ↔ *e*′ pairs preferred to make a conformation hetero-dimerization [62]. Furthermore, biophysical measurements also reveal that repulsive *g* ↔ *e*′ pairs present a more primary role in driving dimerization specificity than attractive *g* ↔ *e*′ pairs [4]. According to the observation of the dimerization properties in our study, the 50 FvbZIP proteins could be classified into 20 types (Supplementary Figure S4 and Table S4).

### 3.7. Expression of *FvbZIP* Genes in Different Organs

**Figure 5:**
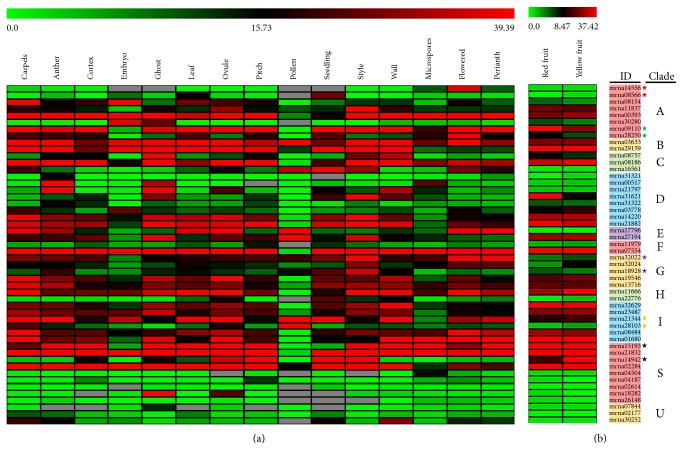
Heatmap of all *FvbZIP* genes expressed in different tissues (a) and red/yellow fruits (b). This heatmap was generated based on the RT-PCR data (normalized) using the MeV software. Green indicates low expression, black indicates intermediate expression, and red indicates high expression.

Many bZIP proteins have been shown to be involved in the processes of plant growth and development [63]. To explore the tissue-specific expression patterns of genomic scale *FvbZIP* genes, the transcriptome data were analyzed to determine those genes expressed in different organs and tissues (Figure 5). *FvbZIP* genes were showed to rank from highest (*mrna21832* in style and yellow fruit) to lowest (*mrna31321* in embryo) according to their differential expression across main tissues. The *FvbZIP* genes clustered in the same clade showed different tissue-specific expression patterns. *mrna15193*, *mrna21832*, *mrna14942*, and *mrna02284* within clade S showed particularly high expression in most organisms, while the others of clade S presented a relatively low expression in all tissues except for *mrna18282* in ghost (refers to the entire seed with its embryo removed) and ovule. The duplicated genes in clades (predicted in the Plant Genome Duplication Database) showed different transcript abundance in specific tissues, such as *mrna32022* and *mrna18928* in flowered and perianth (Figure 5), which is also supported by the duplicated *bZIP* genes in tomato [64]. The divergences in expression profiles between paralogs revealed that some of them may acquire new functions after duplication in the evolutionary process.

As an activator of downstream gene expression, members of clade A bZIP genes share function as important regulatory roles in the ABA signaling pathway, abiotic stresses, and tissues development in *Arabidopsis* [8]. *MdbZIP26* was abundantly expressed in roots, stems, and apical buds [65]. *OsABF2* showed expression in different tissues of rice and induction of drought, salinity, cold, and ABA. In fact, clade A bZIPs often have played key roles not only in seed development but also in fruit maturation [66]. We further analyzed the expression profiles of the 8 clade A *FvbZIP* genes in various organs and fruits using transcriptome data. Firstly, the members show different tissue-specific expression. For example, *mrna00393* and *mrna09110* are moderately expressed in most tissues, while *mrna30280* is more enriched only in flower (Figure 5(a)). Secondly, the respective members of the duplicated *FvbZIP*s pair (*mrna14556* and *mrna08566*, predicted in the Plant Genome Duplication Database) have different pattern specificities. The duplicated pair has a similar expression pattern in each tissue but a different pattern in red and yellow fruits (Figures 5(a) and 5(b)). It is evident that although highly conserved on amino acids sequence, those duplicated genes are differently regulated [67]. Thirdly, the same gene show different expression profiles in different organs. *mrna11837* is expressed at an extremely high level in seedling but very low in majority of other tissues (Figure 5(a)). Overall, the expression of clade A *FvbZIP* genes has different organ specificities, including a functional diversification coupled with the expansion of this gene family in strawberry. At least one clade A bZIP gene (*mrna09110*) is expressed in most investigated strawberry organs. It is consistent with previous studies in *Arabidopsis* and apple [8, 65].

### 3.8. Expression Patterns of *FvbZIP* Genes in Leaves under Drought and Heat Treatments

**Figure 6:**
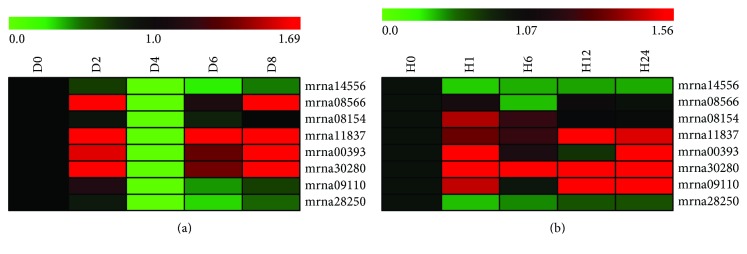
Heatmap of clade A *FvbZIP* genes expressed under drought (a) and heat (b) treatments. This heatmap was generated based on the RT-PCR data (normalized) using the MeV software. Green indicates low expression, black indicates intermediate expression, and red indicates high expression.

We next performed an expression investigation of the clade A *FvbZIP* genes following exposure to drought and heat. A range of expression patterns was observed and some of the genes clearly upregulated, while others were downregulated (Figures 6(a) and 6(b)). As shown in Figure 6(a), for example, *mran00393*, *mrna08566*, *mrna30280*, and *mrna11837* showed a significant upregulation after the plants were treated by drought for 2 days, while all genes in clade A were significantly downregulated at day 4 (Figure 6(a)). These four genes showed a higher expression level than the previous point after rewatering (Figure 6(a)). When exposed to drought, the expression of *mrna14556* and *mrna28250* decreased gradually (Figure 6(a)). Heat treatment caused *mrna30280* transcription levels to increase in all points, and the *mrna30280* transcription level peaked 12 h after the heat treatment was started (Figure 6(a)). Similarly, *mrna00393* showed an obvious increase in expression at 12 h (Figure 6(a)). *mrna14556* and *mrna28250* kept low expression during heat treatment (Figure 6(a)).

It is worth noting that the expression of *mrna11837* in most organs in Figure 5(a) and *mrna30280* in fruit in Figure 5(b) were weak under normal growth conditions but both higher following drought and heat. We conclude that these two genes (*mrna11837* and *mrna30280*) have a different pathway to respond to drought and heat treatments. Drought and heat responses in plants may be a separate pathway, which is consistent with the finding in grape [11]. However, *mrna14556* expression was not detected in any organ, even in response to drought or heat. A similar observation was made in grape, apple, and sorghum with some bZIP genes [65, 68, 69], and we speculate that those genes may be degenerated into a nonfunctional gene, or it is involved in other processes that were not investigated here, such as other abiotic and biotic stresses [68, 69], or they may be expressed at other organs and time points after treatment [65]. This can be supported by the fact that the bZIP *ABF* genes, *ABF1* and *ABF4*, are induced by cold as well as *ABF2* and *ABF3* expression are induced by high salt in vegetative tissues [26, 53]. The constitutive overexpression of *ABF3* in rice and *Arabidopsis* consequently made the transgenic plants more tolerant to stress conditions [70, 71]. Moreover, *OsbZIP23* and *OsbZIP72* play an important role in training drought tolerant rice and resistance to drought treatment by activating ABA signaling [21, 22]. The genes upregulated by stresses was suppressed in mutants of *OsbABF1* under ABA treatment, which indicates that *OsbABF1* is related to abiotic stress responses through ABA pathway in rice. We concluded that the clade A *FvbZIP* genes showed a range of response patterns when exposed to drought and heat stresses, revealing that they may respond to stress signaling, which is also observed in *Arabidopsis*, apple, and grape [8, 65, 68].

## 4. Conclusion

We performed genome-wide analysis of the strawberry bZIP transcription factor family and conducted a detailed investigation of their evolutionary relationship, structural feature, and organization. In addition, we characterized the expression of some clade A genes following drought and heat treatment. These results may prove useful in developing strategies for the further improvement of stress tolerance in strawberry.

## Supplementary Material

Figure S1 Phylogenetic analysis (A) and copy number changes (B) of strawberry, Arabidopsis and rice bZIP proteins. In A, an N-J tree was constructed from a sequence alignment of predicted strawberry, Arabidopsis and rice bZIP proteins using MEGA 6.0 software. Number in branches indicae the bootstrap percentage values calculated from 1000 replicates, and only values >50% are shown. The nodes that represent the most recent common ancestral genes before the strawberry, Arabidopsis and rice split are indicated by red circles (bootstrap support >50%). Clades that contain only one species bZIP protein of are strawberry, Arabidopsis and rice indicated by red, green and yellow, respectively. In B, the numbers in circles and rectangles represent the numbers of bZIP genes in extant and ancestral species, respectively. Number on branch with plus and minus symbols represents the numbers of gene gains and losses, respectively. Figure S2 positions and patterns of introns within tha basic-hinge region of the bZIP domains for 50 FvbZIP transcription factors. The intron position is marked in red stripe. The five intron patterns in FvbZIP domain region were represented by a, b, c, d, and e. Figure S3 Classification of FvbZIP proteins based on the alignment of basic and hinge regions. The conserved amino acids in strawberry bZIP proteins are shadowed in red. The first leucine in leucine heptad repeats is numbered +1 and the last amino acid of hinge regions is -1. Some of the functional annotated bZIP proteins in Arabidopsis and rice sharing similar amino acid sequences in the basic and hinge regions are shown as references. The different amino acid residues at -10 and -18 positions like K and I are colored. Figure S4 Amino acid sequences alignments of the leucine zipper regions of FvbZIP proteins. The FvbZIP proteins are categorized into 20 types with similar predicted dimerization properties. The leucine zipper region is divided into heptad (gabcdef) from L0 to L9 to visualize the potential g ↔ e′pairs. Four colors are used to differentiate between different g ↔ e′pairs. Attractive basic-acidic (R↔E and K↔E) are colored green, attractive acidic-basic pairs (E↔R, E↔K, E↔R, and D↔K) are yellow, repulsive basic pairs (K↔K, R↔K, R↔Q, Q↔K and K↔Q) are blue, repulsive acidic pairs (E↔E, E↔D, E↔Q, and Q↔E) are red. If single amino acid at the positions e of g is charged, the residue is colored blue for basic amino acid and red for acidic acid. If the a or d position is charged, it is colored purple. Asparagines at a position are colored gray. The pralines and glycines are bold to indicate a potential bresk in the α-helix. The predicted C-terminal boundary is denoted by the symbol #, other than the natural terminals which are indicated by the symbol ∗. Table S1 Primer sequence information. Table S2 Additional conserved motifs identified from FvbZIP proteins. Table S3 DNA binding site specificity and classification of FvbZIP proteins. Table S4 Summary for the types of the dimerization properties predicted from FvbZIP proteins. Table S5 Transcriptome data of FvbZIP genes used in this study.

## Figures and Tables

**Table 1:** *bZIP* genes identified in strawberry and their detailed information.

ID	Accession number	Chromosome	Number of amino acids	Molecular weight	Theoretical pI	Instability index	Aliphatic index	GRAVY
mrna00393	XP_011468749.1	LG7	320	35828.4	8.79	55.97	67.66	−0.853
mrna00517	XP_004309443.1	LG6	536	60527.7	6.67	67.1	68.51	−0.76
mrna01680	XP_004290316.1	LG2	585	64330.9	5.96	62.76	54.74	−0.909
mrna02177	XP_004289625.1	LG2	304	34175.6	5.05	71.42	66.38	−0.994
mrna02284	XP_004300452.2	LG5	148	17187.2	5.49	60.81	58.65	−0.943
mrna02614	XP_004292766.1	LG2	158	18178.4	8.9	68.19	67.34	−0.852
mrna03633	XP_004299018.1	LG4	711	76012.1	6.28	52.99	71.31	−0.488
mrna03778	XP_004297132.1	LG4	1106	122711.1	7.64	43.21	89.76	−0.056
mrna04187	XP_004304176.1	LG6	219	25024.5	5.63	64.63	65.89	−0.886
mrna04504	XP_004298330.2	LG4	585	64648.6	6.74	44.74	78.62	−0.458
mrna07554	XP_004289872.1	Unanchored	270	29591	6.01	33.29	57.48	−0.776
mrna07844	XP_011460972.1	LG3	324	36127.8	6.36	45.93	74.1	−0.667
mrna08154	XP_004290511.1	LG2	500	53629.2	6.25	54.88	69.92	−0.41
mrna08186	XP_004289896.1	LG2	425	46306.8	6.07	65.47	70.52	−0.723
mrna08484	XP_004291397.1	LG2	534	57868.1	5.95	60.87	61.22	−0.753
mrna08566	XP_004291338.1	LG2	216	23379.1	9.48	55.37	62.96	−0.634
mrna08757	XP_004291263.1	LG2	366	39534.3	5.49	46.89	64.56	−0.655
mrna09110	XP_004291101.1	LG2	487	52096.4	9.32	44.04	62.05	−0.64
mrna11666	XP_004291469.1	Unanchored	166	18034.9	9.56	68.79	70.6	−0.965
mrna11837	XP_004301547.1	LG5	277	29886.4	6.21	52.67	70.83	−0.739
mrna11979	XP_004303083.1	LG6	535	58896.3	8.66	51.21	88.77	−0.393
mrna13716	XP_011465109.1	LG5	889	96685.2	5.97	55.69	72.56	−0.57
mrna14220	XP_004307087.1	LG7	334	37188.7	7.83	55.28	81.92	−0.558
mrna14556	XP_004289074.1	LG1	227	25468.2	7.15	71.85	61.5	−0.899
mrna14942	XP_004287866.1	LG1	157	17845	4.93	44.46	79.49	−0.58
mrna15193	XP_004291844.1	LG2	171	19140.1	6.51	58.36	71.4	−0.794
mrna16561	XP_004302051.1	LG1	426	46739	5.18	63.18	57.98	−0.895
mrna17796	XP_004303124.1	LG6	329	37026.8	5.85	60.67	62.34	−1.004
mrna18282	XP_011467186.1	LG6	162	18593.9	7.08	60.44	84.81	−0.733
mrna18928	XP_004306702.1	LG7	427	45946.1	8.79	55.68	67.73	−0.725
mrna21344	XP_004307733.1	LG7	364	40093.2	5.91	59.84	59.84	−0.881
mrna21797	XP_004291604.1	Unanchored	543	60038	6.52	51.39	72.14	−0.545
mrna21832	XP_004289640.1	LG2	159	17974.9	6.29	66.22	66.98	−0.701
mrna21882	XP_011467317.1	LG6	631	70226	6.66	52.69	78.56	−0.428
mrna22776	XP_011462861.1	LG4	194	22165.9	5.34	53.68	54.74	−1.346
mrna23487	XP_004309615.1	LG4	445	48838.1	6.08	62.25	61.08	−0.783
mrna26148	XP_004301090.1	LG5	114	13507.3	9	46.47	94.04	−0.712
mrna27194	XP_011460752.1	LG3	848	93710.6	6.1	61.08	68.82	−0.549
mrna28103	XP_004296017.2	LG3	426	46283.3	6.35	59.02	56.92	−0.754
mrna28250	XP_004294799.1	LG3	432	46987.6	9.54	49.8	65.46	−0.663
mrna29159	XP_004300086.1	LG5	320	35003.2	4.73	59.96	80.22	−0.443
mrna29546	XP_004300611.1	Unanchored	734	80035.7	5.74	49.93	64.75	−0.662
mrna30252	XP_011466783.1	LG6	532	58072.2	7.49	61	67.74	−0.619
mrna30280	XP_011466768.1	LG6	437	47924.5	8.45	62.09	60.09	−0.778
mrna31321	XP_004301946.1	LG5	471	52672.4	8.48	47.98	67.94	−0.652
mrna31322	XP_011465538.1	LG5	351	39638.4	6.12	58.95	77.92	−0.572
mrna31621	XP_011466109.1	LG1	362	40736.2	6.35	43.26	79.59	−0.439
mrna32022	XP_011463954.1	LG5	262	28695.4	4.8	44.98	68.93	−0.842
mrna32024	XP_004298867.1	LG5	272	29939.3	4.97	47.36	82.1	−0.544
mrna32629	XP_004292529.1	LG2	336	37040.8	6.64	56.65	61.64	−0.838

## Abbreviations

aa: Amino acids
Ala: Alanine
Asn: Asparagine
bZIP: Basic leucine zipper
DOG: Delay of germination
Ile: Isoleucine
Leu: Leucine
MFMR: Multifunctional mosaic region
MRCAs: Most recent common ancestors.
