# Supplementary material for: Genome-Wide Identification of bZIP Family Genes Involved in Drought and Heat Stresses in Strawberry (Fragaria vesca)
Source: Int J Genomics. 2017 Apr 11;2017:3981031. doi: 10.1155/2017/3981031 (PMC5405593; doi:10.1155/2017/3981031)
Supplement: Supplementary file 1 — Figure S1 Phylogenetic analysis (A) and copy number changes (B) of strawberry, Arabidopsis and rice bZIP proteins. In A, an N-J tree was constructed from a sequence alignment of predicted strawberry, Arabidopsis and rice bZIP proteins using MEGA 6.0 software. Number in branches indicae the bootstrap percentage values calculated from 1000 replicates, and only values >50% are shown. The nodes that represent the most recent common ancestral genes before the strawberry, Arabidopsis and rice split are indicated by red circles (bootstrap support >50%). Clades that contain only one species bZIP protein of are strawberry, Arabidopsis and rice indicated by red, green and yellow, respectively. In B, the numbers in circles and rectangles represent the numbers of bZIP genes in extant and ancestral species, respectively. Number on branch with plus and minus symbols represents the numbers of gene gains and losses, respectively. Figure S2 positions and patterns of introns within tha basic-hinge region of the bZIP domains for 50 FvbZIP transcription factors. The intron position is marked in red stripe. The five intron patterns in FvbZIP domain region were represented by a, b, c, d, and e. Figure S3 Classification of FvbZIP proteins based on the alignment of basic and hinge regions. The conserved amino acids in strawberry bZIP proteins are shadowed in red. The first leucine in leucine heptad repeats is numbered +1 and the last amino acid of hinge regions is -1. Some of the functional annotated bZIP proteins in Arabidopsis and rice sharing similar amino acid sequences in the basic and hinge regions are shown as references. The different amino acid residues at -10 and -18 positions like K and I are colored. Figure S4 Amino acid sequences alignments of the leucine zipper regions of FvbZIP proteins. The FvbZIP proteins are categorized into 20 types with similar predicted dimerization properties. The leucine zipper region is divided into heptad (gabcdef) from L0 to L9 to visualize the po [file 3981031.f1.pptx]

## Slide 1
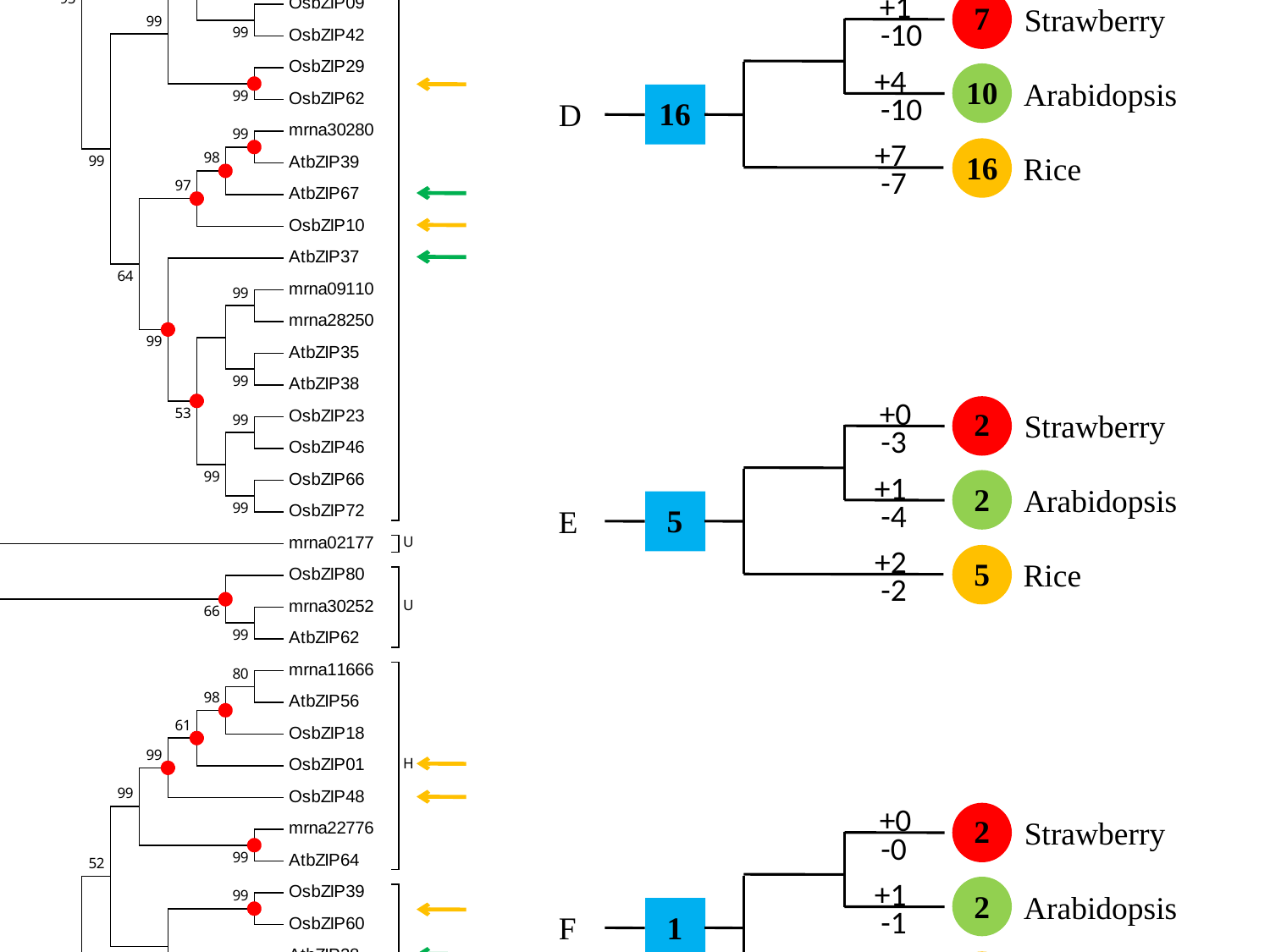

A
B
+2
8
Strawberry
-7
+4
11
Arabidopsis
-6
13
A
+10
17
Rice
-6
+0
2
Strawberry
-2
+1
4
Arabidopsis
-1
4
B
+1
3
Rice
-2
+0
3
Strawberry
-3
+0
4
Arabidopsis
-2
6
C
+3
6
Rice
-3
+1
7
Strawberry
-10
+4
10
Arabidopsis
-10
16
D
+7
16
Rice
-7
+0
2
Strawberry
-3
+1
2
Arabidopsis
-4
5
E
+2
5
Rice
-2
+0
2
Strawberry
-0
+1
2
Arabidopsis
-1
1
F
+1
3
Rice
-0
+1
5
Strawberry
-4
+1
5
Arabidopsis
-4
8
G
+3
7
Rice
-4
+0
2
Strawberry
-2
+0
2
Arabidopsis
-2
4
H
+0
3
Rice
-1
+0
6
Strawberry
-5
+3
9
Arabidopsis
-5
11
I
+4
12
Rice
-3
+3
9
Strawberry
-9
+6
16
Arabidopsis
-5
15
S
+8
12
Rice
-11
