# Supplementary figures and images for: Genome-Wide Identification of bZIP Family Genes Involved in Drought and Heat Stresses in Strawberry (Fragaria vesca)
Source: Int J Genomics. 2017 Apr 11;2017:3981031. doi: 10.1155/2017/3981031 (PMC5405593; doi:10.1155/2017/3981031)

## Slide 1
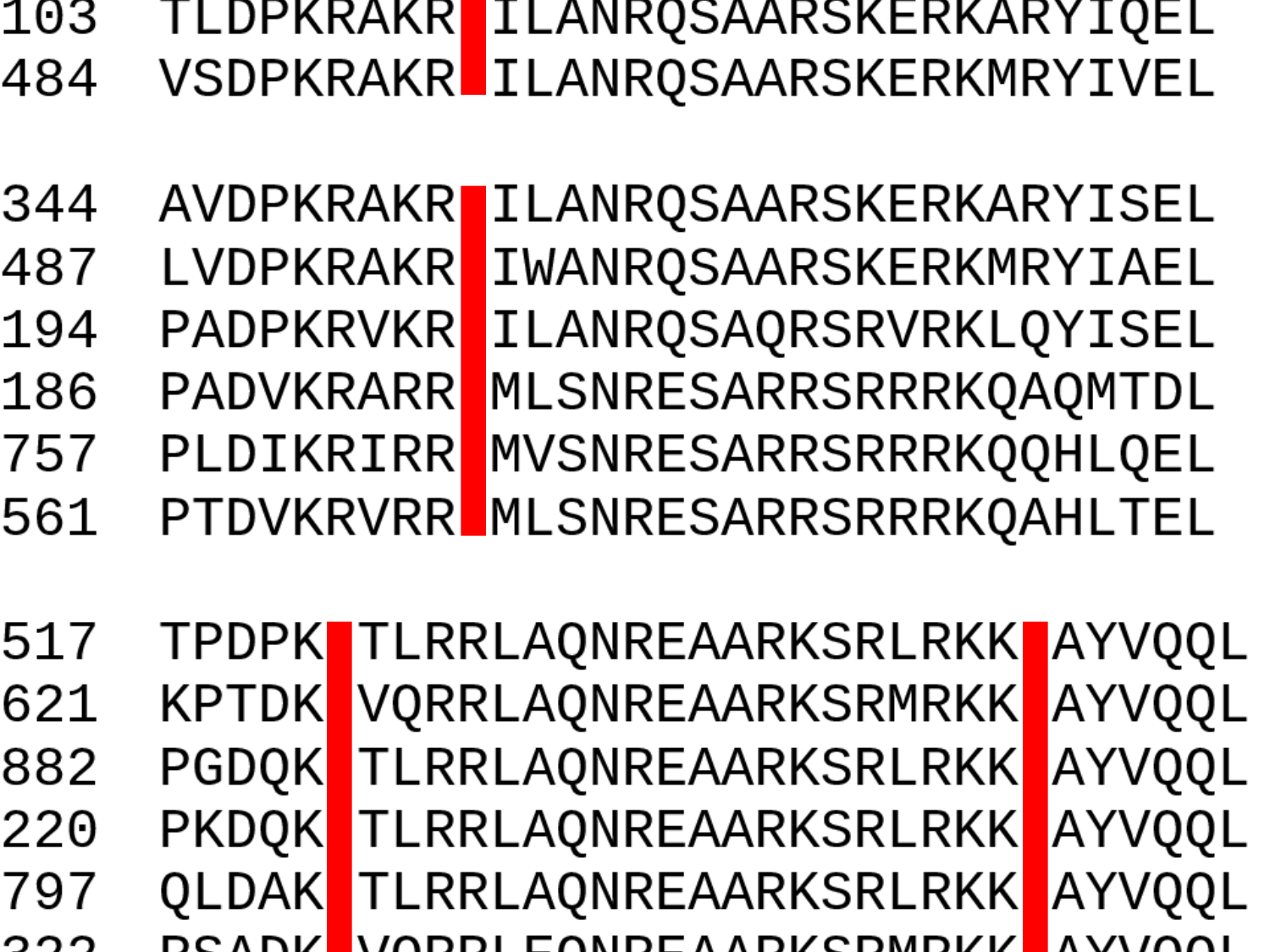

Pattern
FvbZIP ID
Basic Region
Hinge
-30 -25 -20 -15 -10 -5 +1
a
b
c
d
e

Supplement: Supplementary file 2 [file 3981031.f2.pptx]

## Slide 1
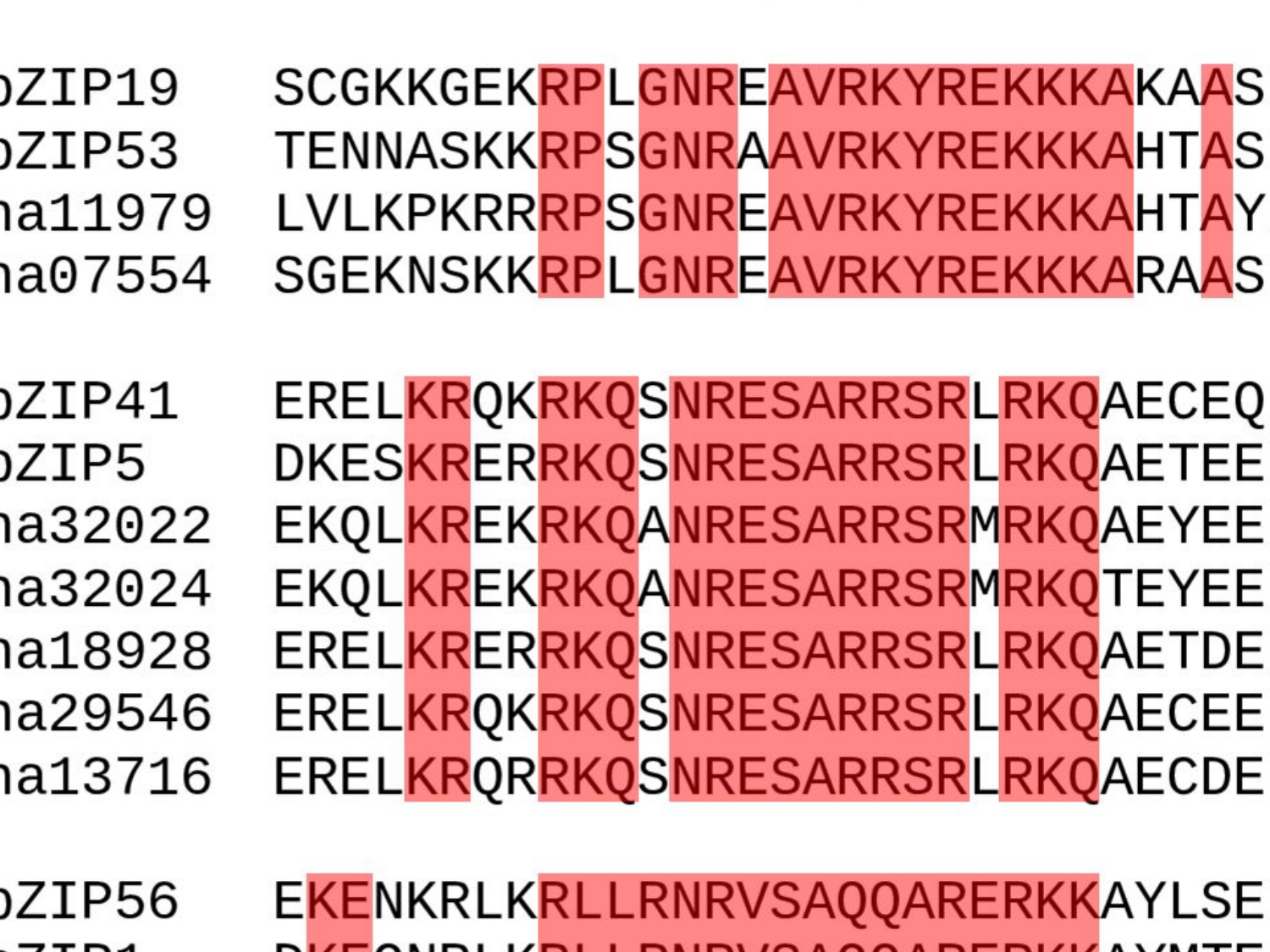

Supplement: Supplementary file 3 [file 3981031.f3.pptx]
