# Supplementary material for: Genome-Wide Identification of bZIP Family Genes Involved in Drought and Heat Stresses in Strawberry (Fragaria vesca)
Source: Int J Genomics. 2017 Apr 11;2017:3981031. doi: 10.1155/2017/3981031 (PMC5405593; doi:10.1155/2017/3981031)
Supplement: Supplementary file 4 [file 3981031.f4.pptx]

## Slide 1
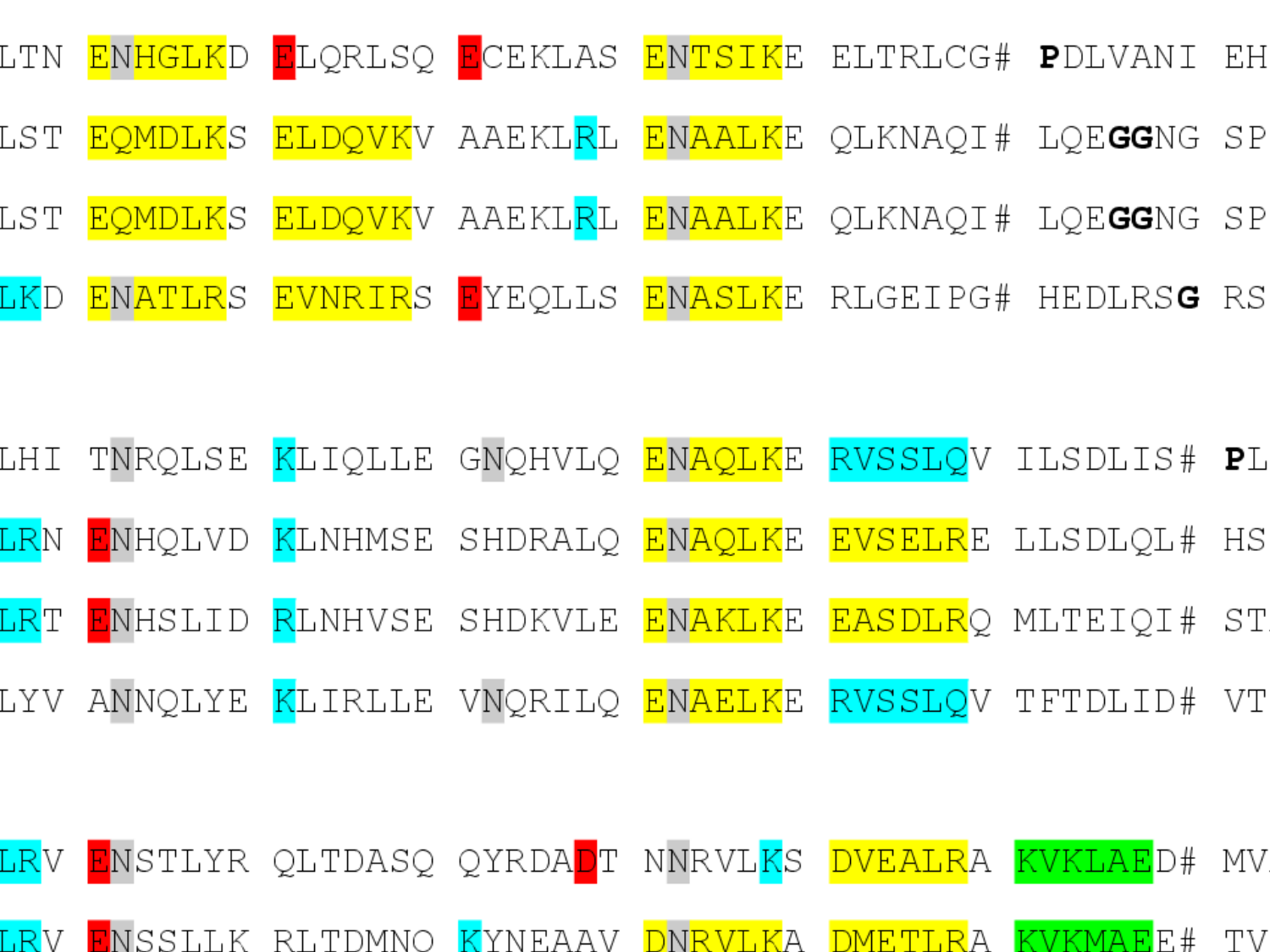

ID Leucine Zipper Region
 L0 L1 L2 L3 L4 L5 L6 L7 L8 L9
gabcdef gabcdef gabcdef gabcdef gabcdef gabcdef gabcdef gabcdef gabcdef gabcdef
1
2
3
4
5
6
7
8
9
10
11
12
13
14
15
16
17
18
19
20
