# Supplementary material for: Genome-Wide Identification of bZIP Family Genes Involved in Drought and Heat Stresses in Strawberry (Fragaria vesca)
Source: Int J Genomics. 2017 Apr 11;2017:3981031. doi: 10.1155/2017/3981031 (PMC5405593; doi:10.1155/2017/3981031)
Supplement: Supplementary file 5 [file 3981031.f5.docx]

### Genome-wide identification of *bZIP* family genes involved in drought and heat stresses in strawberry (*Fragaria vesca*)

**Xiao-Long Wang^1, 2^, Xinlu Chen^2^, Tian-Bao Yang^3^, Qunkang Cheng^4^, Zong-Ming Cheng^1, 2^**

^1^ College of Horticulture, Nanjing Agricultural University, Nanjing 210095, China

^2^ Department of Plant Sciences, University of Tennessee, Knoxville 37996-4560, US

^3^ Food Quality Laboratory, Beltsville Agricultural Research Center, Agricultural Research Service, United States Department of Agriculture, Beltsville, MD 20705, USA

^4^ Department of Entomology and Plant Pathology, University of Tennessee, Knoxville 37996-4560, USA

^*^Corresponding author (E-mail: zmc@njau.edu.cn; Telephone, 86-25-84396055, or zcheng@utk.edu, 865-974-7961)

**Supplementary information**

**Figure S1** **Phylogenetic analysis (A) and copy number changes (B) of strawberry, *Arabidopsis* and rice bZIP proteins.** In **A**, an N-J tree was constructed from a sequence alignment of predicted strawberry, *Arabidopsis* and rice bZIP proteins using MEGA 6.0 software. Number in branches indicae the bootstrap percentage values calculated from 1000 replicates, and only values >50% are shown. The nodes that represent the most recent common ancestral genes before the strawberry, *Arabidopsis* and rice split are indicated by red circles (bootstrap support >50%). Clades that contain only one species bZIP protein of are strawberry, *Arabidopsis* and rice indicated by red, green and yellow, respectively. In **B**, the numbers in circles and rectangles represent the numbers of *bZIP* genes in extant and ancestral species, respectively. Number on branch with plus and minus symbols represents the numbers of gene gains and losses, respectively.

**Figure S2 positions and patterns of introns within tha basic-hinge region of the bZIP domains for 50 FvbZIP transcription factors.** The intron position is marked in red stripe. The five intron patterns in FvbZIP domain region were represented by a, b, c, d, and e.

**Figure S3 Classification of FvbZIP proteins based on the alignment of basic and hinge regions.** The conserved amino acids in strawberry bZIP proteins are shadowed in red. The first leucine in leucine heptad repeats is numbered +1 and the last amino acid of hinge regions is -1. Some of the functional annotated bZIP proteins in *Arabidopsis* and rice sharing similar amino acid sequences in the basic and hinge regions are shown as references. The different amino acid residues at -10 and -18 positions like K and I are colored.

**Figure S4 Amino acid sequences alignments of the leucine zipper regions of FvbZIP proteins.** The FvbZIP proteins are categorized into 20 types with similar predicted dimerization properties. The leucine zipper region is divided into heptad (*gabcdef*) from L0 to L9 to visualize the potential *g* ↔ *e*′pairs. Four colors are used to differentiate between different *g* ↔ *e*′pairs. Attractive basic-acidic (R↔E and K↔E) are colored green, attractive acidic-basic pairs (E↔R, E↔K, E↔R, and D↔K) are yellow, repulsive basic pairs (K↔K, R↔K, R↔Q, Q↔K and K↔Q) are blue, repulsive acidic pairs (E↔E, E↔D, E↔Q, and Q↔E) are red. If single amino acid at the positions *e* of *g* is charged, the residue is colored blue for basic amino acid and red for acidic acid. If the *a* or *d* position is charged, it is colored purple. Asparagines at *a* position are colored gray. The pralines and glycines are bold to indicate a potential bresk in the α-helix. The predicted C-terminal boundary is denoted by the symbol #, other than the natural terminals which are indicated by the symbol *.

**Table S1 Primer sequence information**

**Table S2 Additional conserved motifs identified from FvbZIP proteins**

**Table S3 DNA binding site specificity and classification of FvbZIP proteins**

**Table S4 Summary for the types of the dimerization properties predicted from FvbZIP proteins**

**Table S5 Transcriptome data of *FvbZIP* genes used in this study**

**Table S1**

| **ID** | **Sense** | **AntiSense** |
| --- | --- | --- |
| mrna00393 | TAGCATCACAGTTCCCACAAA | CATGCATCGGAGGTGGTATAG |
| mrna08154 | GTGGTGGTGTAGTGTCATCTTC | GCATGCCACCACCTTTATTTG |
| mrna08566 | CTTCCGGCTCCGATCATTT | AGGGTTTGGTTTCGCTAAGT |
| mrna09110 | CTGGAGTTGTGAGAGAAGATGG | GCTGAAACCCGAATCCTACA |
| mrna11837 | CGCGACTCCTCTATATGTTCTC | GGCTGGTCGTTGTAGATGTT |
| mrna14556 | ATGGAGGAGGTCTGGAAAGA | CAAAGGGCCTAGCGAGAAA |
| mrna28250 | ATGTAAATGGTGGGAAGCTAGG | CAAGTCCTCCCATAGTGTTCTG |
| mrna30280 | CCAACATGGTATGGGTATGGG | CCTTGCAGCAGACTCTCTATTC |
| Fv18S | ACCGTTGATTCGCACAATTGGTCATCG | TACTGCGGGTCGGCAATCGGACG |

**Table S2**

| **Motif** | **Width** | **E-value** | **Consensus sequence** |
| --- | --- | --- | --- |
| Motif 1 | 48 | 1.8E-914 | D[EP][KR][RK]Q[KR]R[MLI][LI][SA]NRE[SA]A[RA][RK]SR[EM]RK[QK]A[YH][VIL]QELEX[KS]VXKL[QR]TENX[EQ]LSR[QE]LT |
| Motif 2 | 50 | 2.80E-157 | LRI[LV]VD[GN][GV][LIM][AS]HYDE[IL]FR[LM]K[GS][TV]AAKADVF[HY][LI]LSGMWKT[PS]AERCF[ML]W[ILM]GGFR |
| Motif 3 | 50 | 4.90E-147 | F[VI]RQAD[NH]LRQQTL[QH]Q[ML][HS]RILTTRQ[AS]AR[ACG]LL[AV][IL][GN][ED]YF[SQ]RLRALSSLW[LMT]ARP |
| Motif 4 | 50 | 6.00E-113 | [LF][LF][QD][RH][DQ][TSR][TL]GL[NTS][NSV][ED]N[SNT][EA]LK[FILQ]R[LI][QA]A[ML][EA]Q[QD][AK][QL][LF][KR]DA[LH][NQ][ED]AL[KT][KE]E[VI][EQ]RL[KR][ILQ][ALV][TY][GH][QE] |
| Motif 5 | 50 | 2.30E-101 | SEL[LI]K[IL]LVNQLEPLT[ED]QQ[LV][ML][GD]I[CY][NS]L[QK]QSSQQAEDAL[ST]QG[ML][ED][AK]LQQ[ST]L[AS][DE]T |
| Motif 6 | 42 | 3.60E-67 | [GAY]H[GS]N[IS][GS][SN]GA[AL][AT]F[DE][MV]EY[AG][RH]W[LV][ED][ED][HQ][HN]R[QL][IM][NS]ELR[AST]A[VL][QN][SAE]H[AL]S[DE][NI]E |
| Motif 7 | 47 | 1.90E-40 | L[AGT]R[EQ][ANST]S[IV]Y[SN]LT[FL]DE[FLV]Q[NH][TQS][LM][GC][GDE][LNP]GK[DNP][FL][GS]SMN[ML]DE[LF]L[KN][SN][IV]W[ST]AE[EA][NT]Q[TAG][IM] |
| Motif 8 | 30 | 1.20E-25 | RQ[PQ]TLGEMTLE[DE]FL[VA][RK]AG[VA]VREDD[QV]KXXX[GLP] |
| Motif 9 | 44 | 7.20E-24 | [DI]T[NS]Q[HK]YM[NE][AL]E[AV][ED]N[RS]VL[KR]A[QD][MV][AE][ET]L[RST][AN][RK][LV][KQ][SM][LA][EN][ED]IVKR[IL][NT]G[NT][NS][GP][LG][FLN] |
| Motif 10 | 38 | 7.70E-18 | [PH][HA]P[YH][MP][WY][GM][AVW][QG][HP][PI][MQ][MPT][PM][MPY][GSY][GPT][PTY][PG][AHV]PY[APV]A[IM]Y[PS][HP]G[GS][VL]YAHP[AGS][MV][PV] |
| Motif 11 | 30 | 7.30E-15 | [SA][LPST][GDS][SNP][SGL][GS][TMS][SGP][GPS][ND][VM]A[ND]YMGQMA[MIL]AM[GN]KL[GAS]TL[EQ][GN] |
| Motif 12 | 30 | 1.30E-14 | Q[LP][SP]LQRQG[SG]L[TLS]L[PS][AR][TAP]LS[KQ]KTVDEVW[KR]E[IL]V[AR] |

**Table S3**

| **Group** | **No. of members** | **Characteristic features** | **Putatve binding site** | **Known binding sites** |
| --- | --- | --- | --- | --- |
| A | 8 | Conseved motifs MIK in the basic region and QAY in the hinge region (except mrna00393) | ABREs with the core ACGT or others containing GCGT or AAGT | CACGTGG/tC, CGCGTG for ABF1[[1](#_ENREF_1)], TRAB1/ OsbZIP66[[2](#_ENREF_2)] Tobacco TGA1b[[3](#_ENREF_3), [4](#_ENREF_4)] and ZmbZIP72[[5](#_ENREF_5)] |
| B | 2 | Key residues in the basic region RNR(/K)E(/D)S(/A)Ax2SR | G- and C-boxes with equal affinity | Tobacco TGA1b[[3](#_ENREF_3), [4](#_ENREF_4)] |
| C | 3 | Specific hinge region sequence QA(/Q)H(/Q)L(/M)T(/Q)E(/D) | Hybrid ACGT elements like G/C,G/A,C/G boxes | GTGAGTCAT for barley BLZ1 and BLZ2[[6](#_ENREF_6), [7](#_ENREF_7)], Antirrhinum (AmbZIP910)[[8](#_ENREF_8)], and GATGAPyPuTGPu for Opaque2[[9](#_ENREF_9)] ocs elements for OBF1 |
| D | 8 | Conserved residues in positions -21 (L/M), -20 (A/E/I), -19 (Q/K),-18 (N),-15 (A/S),-14 (A), -12 (K/R),-11 (S), and -10 (R). Possess a K(/Q)AYV(/T)Q(/N)Q hinge sequence specific to CBFs | GCC binding C-box sequence | TGACGt/g for tobacco TGA1a [[10](#_ENREF_10)], 20 bp ocs-element consensus sequence for OBF3.1 and OBF3.2[[11](#_ENREF_11)] |
| E | 2 | Basic region has A residue at -19 position and hinge region has a conserved QYISE sequence | Relaxed specificity or may bind to other unknown sequences | AtbZIP34 and AtbZIP61[[12](#_ENREF_12)] |
| F | 2 | Conserved residue in position -15 (A) specific to CBFs | C-box elements preferentially | Unknown |
| G | 5 | Conserved residues in positions -18 (N),-15 (S), -14 (A), -11 (S), -10 (R) and has RKQS(/A) conserved sequence in the basic region. Have a A(/T)EC(/T/Y)E(/D)E hinge sequence specific to GBFs | G-box and/or G-box-like sequences | GCCACGTGGC for GBF1, GBF2 and 3[[13](#_ENREF_13)]; AtbZIP16 and AtbZIP68: G-box > Hex > C-box > As-1[[14](#_ENREF_14)] ,G-box containing sequences for ZmGBF1[[15](#_ENREF_15)] |
| H | 2 | NR(/H)VSAQQAR sequence in their basic region | TGACGT-containing Sequences; some G-box-like sequences | Soybean STF1[[16](#_ENREF_16)] ACACGTGG for HY5[[17](#_ENREF_17)] |
| I | 6 | Conserved Lys substitution at -10 position of the basic region instead of Arg | Sequences other than those containing a palindromic ACGT core | TCCAGCTTGA, TCCAACTTGGA for tobacco RSG[[18](#_ENREF_18)]; GCTCCGTTG for tomato VSF-1[[19](#_ENREF_19)] |
| S | 9 | Conserved residues in positions-18 (N),-15 (S), -14 (A),-11 (S), and -10 (R). | TGACGT G-containing | TGACGT G for snapdragon bZIP910/bZIP911[[8](#_ENREF_8)], Ocs enhancer OCSBF-1[[20](#_ENREF_20)]; Wheat histone H3 promoter and the G-box sequence and Adhl promoter for mlip15[[21](#_ENREF_21)] |
| U | 3 | Hydrophobic Ile residue at position -10 instead of Arg/Lys  (except mrna07844 and mrna02177) | Might not be able to bind DNA or else possess a uniquely different DNA-binding specificity | Corresponds to OsZIP-2a reported earlier[[22](#_ENREF_22)] |

**References**

1. Choi H-i, Hong J-h, Ha J-o, Kang J-y, Kim SY: ABFs, a family of ABA-responsive element binding factors. *Journal of Biological Chemistry* 2000, 275(3):1723-1730.

2. Hobo T, Kowyama Y, Hattori T: A bZIP factor, TRAB1, interacts with VP1 and mediates abscisic acid-induced transcription. *Proceedings of the National Academy of Sciences* 1999, 96(26):15348-15353.

3. Katagiri F, Lam E, Chua N-H: Two tobacco DNA-binding proteins with homology to the nuclear factor CREB. 1989.

4. Niu X, Renshaw-Gegg L, Miller L, Guiltinan MJ: Bipartite determinants of DNA-binding specificity of plant basic leucine zipper proteins. *Plant molecular biology* 1999, 41(1):1-13.

5. Wei K, Chen J, Wang Y, Chen Y, Chen S, Lin Y, Pan S, Zhong X, Xie D: Genome-wide analysis of bZIP-encoding genes in maize. *DNA research* 2012, 19(6):463-476.

6. Barley B: a seed-specific bZIP protein that interacts with BLZ1 in vivo and activates transcription from the GCN4-like motif of B-hordein promoters in barley endosperm. *J Biol Chem*, 274.

7. Barley B: a bZIP transcriptional activator that interacts with endosperm-specific gene promoters. *Plant J*, 13:629640.

8. Martínez‐García JF, Moyano E, Alcocer MJ, Martin C: Two bZIP proteins from Antirrhinum flowers preferentially bind a hybrid C‐box/G‐box motif and help to define a new sub‐family of bZIP transcription factors. *The Plant Journal* 1998, 13(4):489-505.

9. Lohmer S, Maddaloni M, Motto M, Di Fonzo N, Hartings H, Salamini F, Thompson RD: The maize regulatory locus Opaque-2 encodes a DNA-binding protein which activates the transcription of the b-32 gene. *The EMBO journal* 1991, 10(3):617.

10. Lam E, Lam YK-P: Binding site requirements and differential representation of TGA factors in nuclear ASF-1 activity. *Nucleic acids research* 1995, 23(18):3778-3785.

11. Foley RC, Grossman C, Ellis JG, Llewellyn DJ, Dennis ES, Peacock WJ, Singh KB: Isolation of a maize bZIP protein subfamily: candidates for the ocs‐element transcription factor. *The Plant Journal* 1993, 3(5):669-679.

12. Shen H, Cao K, Wang X: A conserved proline residue in the leucine zipper region of AtbZIP34 and AtbZIP61 in Arabidopsis thaliana interferes with the formation of homodimer. *Biochemical and biophysical research communications* 2007, 362(2):425-430.

13. Schindler U, Menkens AE, Beckmann H, Ecker JR, Cashmore AR: Heterodimerization between light-regulated and ubiquitously expressed Arabidopsis GBF bZIP proteins. *The EMBO journal* 1992, 11(4):1261.

14. Shen H, Cao K, Wang X: AtbZIP16 and AtbZIP68, two new members of GBFs, can interact with other G group bZIPs in Arabidopsis thaliana. *BMB reports* 2008, 41(2):132-138.

15. Vetten NC, Ferl RJ: Characterization of a maize G‐box binding factor that is induced by hypoxia. *The Plant Journal* 1995, 7(4):589-601.

16. Cheong YH, Yoo CM, Park JM, Ryu GR, Goekjian VH, Nagao RT, Key JL, Cho MJ, Hong JC: STF1 is a novel TGACG‐binding factor with a zinc‐finger motif and a bZIP domain which heterodimerizes with GBF proteins. *The Plant Journal* 1998, 15(2):199-209.

17. Chattopadhyay S, Ang L-H, Puente P, Deng X-W, Wei N: Arabidopsis bZIP protein HY5 directly interacts with light-responsive promoters in mediating light control of gene expression. *The Plant Cell* 1998, 10(5):673-683.

18. Fukazawa J, Sakai T, Ishida S, Yamaguchi I, Kamiya Y, Takahashi Y: Repression of shoot growth, a bZIP transcriptional activator, regulates cell elongation by controlling the level of gibberellins. *The Plant Cell* 2000, 12(6):901-915.

19. Ringli C, Keller B: Specific interaction of the tomato bZIP transcription factor VSF-1 with a non-palindromic DNA sequence that controls vascular gene expression. *Plant molecular biology* 1998, 37(6):977-988.

20. Singh K, Dennis ES, Ellis JG, Llewellyn DJ, Tokuhisa JG, Wahleithner JA, Peacock WJ: OCSBF-1, a maize ocs enhancer binding factor: isolation and expression during development. *The Plant Cell* 1990, 2(9):891-903.

21. Kusano T, Berberich T, Harada M, Suzuki N, Sugawara K: A maize DNA-binding factor with a bZIP motif is induced by low temperature. *Molecular and General Genetics MGG* 1995, 248(5):507-517.

22. Nantel A, Quatrano RS: Characterization of three rice basic/leucine zipper factors, including two inhibitors of EmBP-1 DNA binding activity. *Journal of Biological Chemistry* 1996, 271(49):31296-31305.

**Table S4**

| **Type** | **Members** | **Number of members** | **Heptad with N at a position** | **Length in heptads** | **Comments** |
| --- | --- | --- | --- | --- | --- |
| 1 | mrna14220 | 7 | - | 2, 3 | Absence of attractive g↔e′ interactions as well as presence of charged residues in a position indicate destabilization of homo-dimers. |
|  | mrna00517 |  |  |  |  |
|  | mrna21797 |  |  |  |  |
|  | mrna03778 |  |  |  |  |
|  | mrna21882 |  |  |  |  |
|  | mrna31621 |  |  |  |  |
|  | mrna31322 |  |  |  |  |
| 2 | mrna14556 | 1 | L2 | 3 | Presence of attractive g↔e′ interactions in the 1st heptad and N in a position of 2nd heptad, as well as lack of any repulsive interactions will favor strongly homo-dimerization between the same RcbZIP proteins or within the subfamily. |
| 3 | mrna08566 | 1 | L2 | 3 | Ns in a position of 2nd heptad and an attractive g↔e′ interaction in the 2nd heptad indicate homo-dimerization. While the presence of one repulsive g↔e′ interactions of 1st heptad imply the formation of hetero-dimerization. |
| 4 | mrna31321 | 2 | L2 | 3 | N in a position of 2nd heptad and an attractive g↔e′ interaction in the 1st heptad indicate homo-dimerization. Repulsive and incomplete g↔e′ interactions may support hetero-dimerization with other similar RcbZIP proteins. |
|  | mrna00393 |  |  |  |  |
| 5 | mrna11666 | 3 | L2, L4 | 4 | An attractive g↔e′pair and presence of N in a position of 2nd and/or 4th heptad favor dimerization with itself and the other members of the subfamily. Repulsive g↔e′interactions and incomplete electrostatic pairs in the 1st and 3rd heptads prevents hetero-dimerization. |
|  | mrna22776 |  |  |  |  |
|  | mrna03633 |  |  |  |  |
| 6 | mrna29159 | 1 | L4 | 4 | Two attractive g↔e′ interactions in the 1st and 4th heptads and presence of N in a position of 4th heptad can be beneficial to homo-dimerization formation. Presence of incomplete g↔e′ pairs in 2nd heptad may favor hetero-dimerization. |
| 7 | mrna28250 | 2 | L2 | 4, 5 | N in a position of 2nd heptad and the 1st or 3rd heptad have attractive g↔e′ pairs should encourage homo-dimerization and/or dimerization within the subfamily. |
|  | mrna11837 |  |  |  |  |
| 8 | mrna08154 | 1 | L2 | 5 | Four incomplete g↔e′ pairs favor hetero-dimerization. |
| 9 | mrna07554 | 1 | L2 | 6 | Potential to be homo-dimerization for the presence of N in a position and an attractive g↔e′pairs of 2nd heptad. Incomplete g↔e′ interactions may form hetero-dimerization with the similar subfamilies. |
| 10 | mrna02177 | 6 | L2, L5 | 6 | Two attractive g↔e′pairs and N at a position of 2nd and 5th heptad promote homo-dimer. Incomplete g↔e′pairs may favor hetero-dimerization. |
|  | mrna18928 |  |  |  |  |
|  | mrna29546 |  |  |  |  |
|  | mrna32022 |  |  |  |  |
|  | mrna32024 |  |  |  |  |
|  | mrna13716 |  |  |  |  |
| 11 | mrna18282 | 4 | L2, L5 | 7 | Stabilization of homo-dimers by N at a position of 2nd and 5th heptads and attractive g↔e′ pairs in the 5th and 6th heptads. A repulsive g↔e′ interaction in the 1st or 6th heptads and/or incomplete g↔e′ interactions might support hetero-dimerization. |
|  | mrna04187 |  |  |  |  |
|  | mrna04504 |  |  |  |  |
|  | mrna26148 |  |  |  |  |
| 12 | mrna08757 | 3 | L2, L5 | 7 | Both 6th and 7th heptads have attractive g↔e′ interactions and N is present in 2nd and 5th heptads a position supporting homo-dimerization. However, presence of a repulsive g↔e′ pair in 1st heptad and incomplete g↔e′ pairs may favor hetero-dimerization. |
|  | mrna16561 |  |  |  |  |
|  | mrna08186 |  |  |  |  |
| 13 | mrna11979 | 1 | L2 | 8 | Presence of an attractive g↔e′pair in a position of 8th heptad favor dimerization with itself and the other members of the subfamily. Repulsive g↔e′interaction in the 1st heptadand and incomplete electrostatic pairs prevents hetero-dimerization. |
| 14 | mrna15193 | 3 | L2, L5 | 8 | Incomplete and repulsive g↔e′pairs in 1st heptad suggest the probability of hetero-dimerization. Homo-dimerization also can be formed by an attractive g↔e′pair in 5th heptad and Ns in a position of 5th and 7th heptad. |
|  | mrna14942 |  |  |  |  |
|  | mrna02284 |  |  |  |  |
| 15 | mrna30252 | 1 | L2, L5 | 8 | Ns in a position of 2nd and 5th heptad and an attractive g↔e′ interaction in the 2nd heptad indicate homo-dimerization. While the presence of one repulsive g↔e′ interactions of 4th heptad imply the formation of hetero-dimerization. |
| 16 | mrna21832 | 1 | L2 | 9 | Three repulsive g↔e′ interactions and lacking of any attractive pairs may drive hetero-dimerization. |
| 17 | mrna30280 | 2 | L2 | 9 | Two attractive g↔e′pairs favor dimerization. One repulsive and incomplete g↔e′interactions may drive hetero-dimerization. |
|  | mrna09110 |  |  |  |  |
| 18 | mrna02614 | 1 | L2, L5 | 9 | Ns in 2nd and 5th heptad a positions and an repulsive g↔e′interaction in the 5th heptad favor hetero-dimerization. Occurrence of two attractive g↔e′pairs in 1st and 2nd heptads should promote homo-dimerization. |
| 19 | mrna27194 | 3 | L5 | 9 | Homo-dimers could be formed because of attractive g↔e′ pairs in 2nd, 5th and 9th heptads along with N in a position of 5th heptad. Hetero-dimers could also be stabilized due to two repulsive g↔e′ pairs in the 7th and 8th heptads as well as incomplete g↔e′ pairs. |
|  | mrna17796 |  |  |  |  |
|  | mrna07844 |  |  |  |  |
| 20 | mrna21344 | 6 | L5, L8 | 9 | Two Ns in a position of 5th and 8th heptads and three attractive g↔e′pairs favor dimerization. Incomplete and one repulsive g↔e′ |
|  | mrna08484 |  |  |  |  |
|  | mrna28103 |  |  |  | interaction may drive hetero-dimerization. |
|  | mrna32629 |  |  |  |  |
|  | mrna01680 |  |  |  |  |
|  | mrna23487 |  |  |  |  |

**Table S5**

|  | | | | | | | | |  |
| --- | --- | --- | --- | --- | --- | --- | --- | --- | --- |
|  | Carpels | Anther | Cortex | Embryo | Ghost | Leaf | Ovule | Pitch |  |
| mrna00393 | 77.89 | 50.52 | 42.59 | 116.30 | 80.49 | 35.67 | 66.29 | 42.41 |  |
| mrna00517 | 0.94 | 71.20 | 0.05 | 0.43 | 32.52 | 0.05 | 0.10 |  |  |
| mrna01680 | 57.53 | 26.28 | 28.93 | 9.14 | 37.56 | 15.75 | 100.57 | 22.60 |  |
| mrna02177 | 10.45 | 8.88 | 3.63 | 2.59 | 0.56 | 7.58 | 1.83 | 3.29 |  |
| mrna02284 | 237.19 | 110.98 | 61.07 | 125.28 | 84.78 | 117.59 | 271.77 | 66.91 |  |
| mrna02614 | 0.62 | 0.31 | 0.53 |  | 1.14 | 0.92 | 0.18 | 0.90 |  |
| mrna03633 | 83.57 | 62.18 | 26.79 | 38.24 | 54.13 | 55.94 | 93.18 | 34.27 |  |
| mrna03778 | 21.38 | 12.69 | 5.16 | 25.12 | 12.30 | 8.97 | 11.53 | 8.65 |  |
| mrna04187 | 0.54 | 0.66 | 6.98 | 0.27 | 0.18 | 0.61 | 0.42 | 12.13 |  |
| mrna04504 | 0.89 | 0.18 | 0.36 | 0.08 | 0.22 | 0.31 |  | 1.24 |  |
| mrna07554 | 73.01 | 80.02 | 70.59 | 55.94 | 56.26 | 65.99 | 65.00 | 72.74 |  |
| mrna07844 | 0.88 |  |  | 1.34 | 13.17 |  | 2.46 |  |  |
| mrna08154 | 42.02 | 16.18 | 21.06 | 39.76 | 8.24 | 26.67 | 22.50 | 18.78 |  |
| mrna08186 | 46.68 | 41.99 | 46.14 | 18.12 | 44.45 | 29.19 | 58.73 | 46.59 |  |
| mrna08484 | 40.22 | 24.55 | 24.65 | 15.69 | 20.83 | 29.45 | 33.28 | 22.94 |  |
| mrna08566 | 0.44 | 3.55 | 0.17 | 8.36 | 2.46 | 16.40 | 1.91 | 0.62 |  |
| mrna08757 | 6.21 | 16.70 | 16.55 | 0.73 | 105.33 | 6.14 | 10.61 | 18.04 |  |
| mrna09110 | 83.87 | 37.24 | 69.97 | 4.29 | 37.62 | 49.82 | 45.90 | 79.20 |  |
| mrna11666 | 53.75 | 25.63 | 26.82 | 26.61 | 36.58 | 43.21 | 88.44 | 27.05 |  |
| mrna11837 | 19.83 | 17.35 | 13.69 | 2.95 | 18.04 | 12.60 | 30.49 | 14.68 |  |
| mrna11979 | 5.21 | 4.18 | 9.78 | 4.89 | 5.24 | 10.36 | 5.43 | 14.29 |  |
| mrna13716 | 37.52 | 33.02 | 18.49 | 17.52 | 27.91 | 24.96 | 28.10 | 18.06 |  |
| mrna14220 | 38.64 | 29.58 | 25.59 | 14.68 | 34.79 | 32.44 | 37.15 | 27.67 |  |
| mrna14556 | 2.39 | 0.88 | 0.60 |  |  | 0.27 | 0.08 | 0.59 |  |
| mrna14942 | 3.60 | 2.89 | 17.17 | 0.70 | 18.00 | 10.25 | 34.98 | 46.53 |  |
| mrna15193 | 25.42 | 214.25 | 857.33 | 5.09 | 384.89 | 113.76 | 270.47 | 719.23 |  |
| mrna16561 | 22.22 | 8.35 | 2.38 | 0.71 | 2.61 | 9.34 | 17.01 | 7.90 |  |
| mrna17796 | 38.15 | 31.32 | 13.36 | 4.53 | 9.11 | 37.03 | 69.03 | 8.83 |  |
| mrna18282 | 0.90 | 0.67 |  | 1.04 | 172.43 | 0.14 | 24.11 | 0.16 |  |
| mrna18928 | 9.79 | 20.96 | 6.63 | 6.61 | 13.56 | 14.93 | 12.70 | 9.56 |  |
| mrna21344 | 64.42 | 67.93 | 18.71 | 34.80 | 15.90 | 38.67 | 69.61 | 15.07 |  |
| mrna21797 | 4.65 | 73.28 | 0.41 | 0.83 | 40.41 | 1.53 | 22.97 | 3.58 |  |
| mrna21832 | 265.56 | 585.02 | 330.74 | 64.75 | 253.49 | 255.36 | 304.95 | 485.69 |  |
| mrna21882 | 36.54 | 32.22 | 31.59 | 11.47 | 40.12 | 36.26 | 37.27 | 38.76 |  |
| mrna22776 | 1.94 | 1.42 | 1.98 | 1.02 | 1.15 | 8.91 | 18.21 | 0.53 |  |
| mrna23487 | 31.58 | 26.44 | 24.88 | 24.93 | 35.57 | 18.65 | 27.28 | 23.40 |  |
| mrna26148 | 0.39 | 2.38 | 0.25 | 0.24 | 0.52 | 0.22 | 1.32 | 0.30 |  |
| mrna27194 | 21.64 | 21.72 | 25.11 | 6.13 | 37.41 | 12.68 | 25.16 | 21.64 |  |
| mrna28103 | 23.28 | 13.28 | 8.72 | 5.40 | 1.40 | 8.63 | 17.33 | 5.61 |  |
| mrna28250 | 22.22 | 20.76 | 19.44 | 0.39 | 8.61 | 23.05 | 7.78 | 18.71 |  |
| mrna29159 | 111.61 | 62.52 | 26.70 | 45.03 | 51.93 | 44.72 | 266.35 | 38.63 |  |
| mrna29546 | 38.61 | 28.55 | 23.53 | 12.95 | 33.48 | 26.01 | 45.92 | 21.04 |  |
| mrna30252 | 21.56 | 15.73 | 5.04 | 6.58 | 1.95 | 5.35 | 3.91 | 4.10 |  |
| mrna30280 | 0.34 | 0.68 | 0.07 | 37.29 | 25.30 | 0.31 | 0.13 | 0.15 |  |
| mrna31321 | 0.16 | 15.68 | 0.26 | 0.04 | 0.14 | 0.34 | 0.37 | 0.07 |  |
| mrna31322 | 4.15 | 15.74 | 13.11 | 0.27 | 9.20 | 5.61 | 5.31 | 11.43 |  |
| mrna31621 | 3.67 | 10.00 | 13.62 | 0.30 | 29.01 | 5.82 | 5.48 | 12.38 |  |
| mrna32022 | 27.81 | 26.06 | 22.75 | 7.35 | 17.25 | 19.14 | 21.84 | 23.29 |  |
| mrna32024 | 15.99 | 16.17 | 13.37 | 5.71 | 10.76 | 17.54 | 14.37 | 12.86 |  |
| mrna32629 | 27.98 | 26.15 | 19.60 | 12.23 | 30.64 | 25.21 | 30.43 | 24.96 |  |
|  |  |  |  |  |  |  |  |  |  |
|  | Pollen | Seedling | Style | Wall | Microspores | Flowered | Perianth | Red Fruit | Yellow Fruit |
| mrna00393 | 6.90 | 17.47 | 41.16 | 43.88 | 46.94 | 94.62 | 53.92 | 22.73 | 26.25 |
| mrna00517 | 0.16 | 3.40 | 1.39 | 1.31 | 24.15 | 4.41 | 2.81 | 1.55 | 1.76 |
| mrna01680 | 0.36 | 16.68 | 16.00 | 48.24 | 39.65 | 64.82 | 60.64 | 58.02 | 61.26 |
| mrna02177 | 0.37 | 7.12 | 0.92 | 3.71 | 1.87 | 4.44 | 4.12 | 0.25 | 0.07 |
| mrna02284 | 16.87 | 154.54 | 472.60 | 191.36 | 12.65 | 28.42 | 23.37 | 68.83 | 101.57 |
| mrna02614 |  | 2.85 | 0.18 | 0.96 | 0.85 | 1.23 | 0.85 | 0.24 | 0.27 |
| mrna03633 | 45.26 | 26.42 | 64.74 | 53.05 | 26.29 | 57.05 | 29.87 | 22.69 | 23.73 |
| mrna03778 | 0.09 | 6.11 | 7.19 | 10.99 | 4.41 | 17.27 | 9.60 | 8.36 | 14.25 |
| mrna04187 | 0.13 | 0.45 | 0.26 | 0.34 | 4.39 | 6.20 | 3.27 | 0.00 | 0.10 |
| mrna04504 |  | 0.58 |  | 0.10 | 14.72 | 4.54 | 1.67 | 0.00 | 0.00 |
| mrna07554 | 1.80 | 42.16 | 74.23 | 59.85 | 44.29 | 58.23 | 51.69 | 88.54 | 96.14 |
| mrna07844 | 0.09 |  | 0.08 | 0.69 | 1.77 | 2.32 | 0.83 | 0.00 | 0.00 |
| mrna08154 | 0.23 | 7.62 | 12.18 | 12.50 | 3.53 | 16.78 | 9.72 | 5.06 | 3.52 |
| mrna08186 | 1.40 | 22.32 | 59.17 | 43.66 | 28.21 | 35.75 | 32.21 | 32.34 | 56.97 |
| mrna08484 | 0.51 | 20.54 | 27.98 | 25.80 | 23.69 | 52.10 | 34.56 | 27.81 | 24.67 |
| mrna08566 |  | 26.60 | 0.09 | 0.25 | 2.65 | 2.48 | 1.46 | 0.09 | 0.00 |
| mrna08757 | 0.08 | 4.68 | 27.05 | 34.16 | 1.43 | 5.10 | 2.40 | 10.07 | 6.85 |
| mrna09110 | 0.17 | 152.89 | 100.14 | 49.18 | 21.51 | 134.80 | 69.24 | 41.28 | 24.32 |
| mrna11666 | 0.25 | 18.30 | 329.66 | 47.39 | 22.72 | 53.42 | 32.74 | 28.16 | 38.62 |
| mrna11837 | 10.26 | 13.11 | 92.94 | 20.41 | 11.74 | 12.11 | 12.47 | 21.79 | 19.10 |
| mrna11979 |  | 12.79 | 8.43 | 4.89 | 3.68 | 7.80 | 5.46 | 2.38 | 2.93 |
| mrna13716 | 5.17 | 29.47 | 31.37 | 29.81 | 21.04 | 26.92 | 22.34 | 19.68 | 19.06 |
| mrna14220 | 1.52 | 29.56 | 44.88 | 25.92 | 6.23 | 17.87 | 15.72 | 28.42 | 29.26 |
| mrna14556 |  |  | 0.52 | 0.50 | 10.12 | 37.25 | 9.74 | 0.00 | 0.00 |
| mrna14942 | 0.17 | 138.64 | 39.99 | 2.52 | 0.44 | 3.85 | 4.13 | 16.04 | 37.42 |
| mrna15193 | 0.68 | 4.62 | 115.38 | 179.90 | 49.95 | 29.60 | 51.94 | 107.77 | 274.21 |
| mrna16561 | 34.00 | 139.44 | 31.23 | 1.78 | 3.47 | 27.48 | 19.82 | 0.41 | 0.60 |
| mrna17796 | 407.55 | 8.91 | 7.58 | 12.31 | 8.74 | 45.94 | 124.96 | 0.29 | 0.26 |
| mrna18282 |  |  | 2.13 | 2.40 | 1.66 | 1.32 | 1.18 | 0.00 | 0.13 |
| mrna18928 | 14.77 | 24.41 | 35.88 | 16.45 | 3.60 | 8.71 | 6.61 | 5.52 | 4.17 |
| mrna21344 | 463.70 | 49.02 | 17.84 | 39.39 | 7.21 | 31.94 | 35.12 | 12.30 | 15.84 |
| mrna21797 | 0.05 | 16.26 | 10.04 | 32.59 | 13.19 | 3.86 | 6.75 | 1.78 | 1.78 |
| mrna21832 | 2.43 | 111.51 | 1045.02 | 682.67 | 39.92 | 66.17 | 41.96 | 411.42 | 445.93 |
| mrna21882 | 1.80 | 35.88 | 52.72 | 37.76 | 8.79 | 25.78 | 21.29 | 43.08 | 32.01 |
| mrna22776 |  | 24.16 | 12.44 | 16.29 | 1.30 | 5.76 | 7.57 | 0.50 | 1.87 |
| mrna23487 | 5.57 | 17.72 | 29.29 | 27.37 | 7.48 | 17.65 | 15.47 | 24.60 | 26.17 |
| mrna26148 |  |  |  | 0.29 | 3.51 | 3.85 | 2.71 | 0.00 | 0.19 |
| mrna27194 | 206.59 | 14.72 | 36.34 | 16.72 | 13.24 | 9.83 | 8.64 | 26.46 | 28.31 |
| mrna28103 | 199.96 | 4.76 | 10.10 | 10.30 | 7.41 | 10.88 | 9.94 | 1.81 | 2.97 |
| mrna28250 | 0.07 | 23.83 | 8.00 | 10.30 | 19.78 | 41.41 | 16.55 | 8.58 | 5.86 |
| mrna29159 | 11.69 | 96.33 | 69.71 | 105.17 | 8.67 | 11.77 | 10.76 | 53.71 | 44.67 |
| mrna29546 | 0.83 | 25.46 | 24.37 | 32.93 | 10.02 | 25.48 | 15.02 | 18.11 | 21.70 |
| mrna30252 |  | 16.41 | 4.07 | 28.71 | 3.36 | 13.61 | 12.22 | 0.73 | 0.77 |
| mrna30280 |  | 0.36 | 0.07 | 3.74 | 3.24 | 3.85 | 1.54 | 5.70 | 4.07 |
| mrna31321 | 0.39 |  | 0.14 | 0.15 | 0.78 | 1.34 | 6.05 | 0.04 | 0.00 |
| mrna31322 | 0.44 | 1.19 | 7.64 | 1.70 | 5.85 | 4.89 | 3.76 | 5.44 | 4.70 |
| mrna31621 | 0.08 | 21.90 | 48.77 | 24.49 | 10.37 | 4.15 | 3.96 | 52.88 | 8.11 |
| mrna32022 | 21.49 | 27.47 | 40.09 | 20.31 | 22.51 | 48.20 | 43.25 | 4.34 | 5.72 |
| mrna32024 | 6.73 | 9.90 | 28.77 | 12.18 | 15.83 | 25.01 | 25.50 | 3.33 | 9.13 |
| mrna32629 | 0.08 | 24.54 | 31.02 | 51.75 | 11.87 | 17.77 | 15.52 | 340.77 | 430.64 |
|  |  |  |  |  |  |  |  |  |  |
